# Ethanol extract of *Andrographis paniculata* alleviates aluminum-induced neurotoxicity and cognitive impairment through regulating the p62-keap1-Nrf2 pathway

**DOI:** 10.1186/s12906-023-04290-4

**Published:** 2023-12-06

**Authors:** Jianping Ma, Miao Zheng, Xinyue Zhang, Jiaqi Lu, Lili Gu

**Affiliations:** 1 Department of Pharmacy, Affiliated Hospital of Hangzhou Normal University, Hangzhou, China; 2 Key Laboratory of Neuropsychiatric Drug Research of Zhejiang Province, School of Pharmacy (Institute of Materia Medica), Hangzhou Medical College, Hangzhou, Zhejiang 310013 China

**Keywords:** Aluminum, Neuroprotective, Cognitive dysfunction, *Andrographis paniculata*, Andrographolide, Autophagy

## Abstract

**Background:**

Alzheimer’s disease (AD) is the most prevalent neurodegenerative and remains incurable. Aluminum is a potent neurotoxin associated with AD. The main pathological features of AD are extracellular amyloid-β protein deposition and intracellular hyperphosphorylated Tau protein. A body of evidence suggest that oxidative stress and autophagy are involved in the pathogenesis of AD. *Andrographis paniculata* (AP) is a native plant with anti-inflammatory, anti-oxidative stress, and regulation of autophagy properties. AP significantly alleviated cognitive impairments, reduced Aβ deposition and has neuroprotective effect. However, its effects on aluminum-induced AD model have not been studied much. In this study, we investigated whether *AP* protect against aluminum-induced neurotoxicity through regulation of p62-Kelch-like ECH-associated protein 1(Keap1)-Nuclear factor E2 related factor 2 (Nrf2) pathway and activation autophagy in vivo and in vitro.

**Methods:**

UPLC-ESI-qTOF-MS/MS was used to identify the chemical constituents of AP ethanol extract. The mice with cognitive deficit were established by injecting aluminum chloride and D-galactose, and treated with either AP extract (200, 400, or 600 mg/kg/d) or andrographolide (2 mg/kg/2d).The spatial memory ability was detected by Morris water maze, HE staining were used to detect in brain tissue,Oxidative stress indexs and SOD activity in both serum and brain tissue were detected by kit.The expression of p62-Nrf2 pathway proteins were measured via western blotting. Furthermore, the neurotoxicity model was induced by aluminum maltolate (700 µM) in PC12 cells. Following AP and andrographolide treatment, the cell viability was detected. The relevant mRNA and protein expressions were detected in cells transfected with the p62 siRNA.

**Results:**

The main active components of AP included andrographolide, neoandrographolide and deoxyandrographolide as identified. AP and andrographolide significantly improved the spatial memory ability of mice, attenuated pathological changes of hippocampal cells, reduced the level of malondialdehyde, and increased superoxide dismutase activity in serum or brain tissue as compared to model control. In addition, the Nrf2, p62 and LC3B-II proteins expression were increased, and p-Tau and Keap1 proteins were decreased in the hippocampus after AP and andrographolide treatment.Furthermore, AP increased aluminum maltolate-induced cell viability in PC12 cells. Silencing p62 could reverse the upregulation expression of Nrf2 and downregulation of Keap1 and Tau proteins induced by AP in aluminum maltolate-treated cells.

**Conclusions:**

AP had neuroprotective effects against aluminum -induced cognitive dysfunction or cytotoxicity, which was involved in the activation of the p62-keap1-Nrf2 pathway and may develop as therapeutic drugs for the treatment of AD. However, this study has certain limitations, further optimize the protocol or model and study the molecular mechanism of *AP* improving AD.

**Supplementary Information:**

The online version contains supplementary material available at 10.1186/s12906-023-04290-4.

## Background

Alzheimer’s disease (AD) is the most common type of dementia in older people. It is a severe neurodegenerative disease with a high frequency. The main pathological features of AD are the senile plaques which are induced by extracellular amyloid-β (Aβ) protein deposition and tangles of nerve fibers which are induced by intracellular hyperphosphorylated Tau (p-Tau) protein [1]. A body of evidence suggest that oxidative stress and autophagy are involved in the pathogenesis of AD [2]. Aluminum (Al) is a well-known neurotoxic agent, Al has been detected in both senile plaques and NFT-bearing neurons in the brains of AD patients, which suggests roles for this metal in AD [3]. Al binding can enhance Aβ protein penetration of the blood brain barrier [4]. Several studies have demonstrated that Al can induce pathological changes similar to AD including the accumulation of tau and Aβ, neuronal apoptosis, and disruption of iron and calcium homeostasis [5–7]. So Al-induced AD model is an excellent model for screening new anti-AD drugs. A large number of studies have shown that the pathogenesis of Al-induced AD is mainly related to oxidative stress and autophagy dysfunction [8]. Oxidative stress is strongly thought to play a key role in Al neurotoxicity. Previous studies have shown that Al exposure promotes reactive oxygen species (ROS) generation and reduces antioxidant enzyme activity in animal models. In other words, Al exposure induced lipid peroxidation (LPO) and diminished superoxide dismutase (SOD), catalase (CAT), and glutathione peroxidase (GPx) activity [9, 10]. Autophagy dysfunction is involved in the pathogenesis of neurodegenerative disorders. Intriguingly, it was observed that the expression of autophagy-associated proteins, such as autophagy-related genes 5 (Atg5), Beclin-1, and LC3II, was intensely declined in the aluminum chloride-treated rats, while downregulated autophagy process resulted in reduced autophagic clearance of Aβ protein aggregates [8]. Taken together, The pathological mechanism of cognitive impairment induced by Al is closely related to oxidative stress and autophagy dysfunction. Traditional Chinese medicine (TCM) has shown efficacy in treating AD by regulating apoptosis, autophagy, neuro-inflammation, oxidative stress, mitochondrial dysfunction, and other factors [11].

*Andrographis paniculata* (AP) (Burm.f.) Nees is a medicinal plant from the family Acanthaceae that has various pharmacological properties, including anti-inflammatory, anti-oxidative stress and regulation of autophagy and other factors [12]. Pharmacodynamic studies have shown can cross the blood brain barrier distribute into different regions, therefore its pharmacological effects central nervous system begun be revealed recent years [13]. The therapeutic effect of AP on AD has been confirmed in various animal models, such as streptozotocin-induced diabetic rats [14], lipopolysaccharide-induced cognitive deficit [15], scopolamine-induced amnesic rats [16], and even in a drosophila melanogaster model of neurotoxicity [17]. However, the effect of AP on Al-induced AD model has not been reported much. Despite these findings, the exact mechanism of action for AP remains to be fully elucidated. AP active ingredients are derived from biologically active ingredients, mainly diterpene lactones, including andrographolide (Andro), dehydroandrographolide, neoandrographolide, and deoxyandrographolide [18]. In animal studies, andrographolide, the major labdanum diterpene isolated from AP leaves or whole plants, has been shown to be important in the treatment of AD [19]. Andrographolide reduces cognitive impairment, as well as neuritis and oxidative stress, recover the synaptic function, and promote neuron generation [20]. In a previous investigation, we performed a network-based analysis to examine the principal pharmacological pathways that AP employs in the context of AD [21]. Our results revealed that AP engages in the modulation of several pathways [21]. A recent study has revealed that andrographolide is capable of providing substantial protection to PC12 cells against Aβ-induced cytotoxicity. This noteworthy finding has been linked with the activation of nuclear factor-erythroid related factor 2 (Nrf2) and SQSTM1/p62, commonly referred to as p62 [22].

The Nrf2-Kelch-like ECH-associated protein 1 (Keap1) pathway is a crucial mediator that plays a pivotal role in various cellular defense mechanisms. P62 has been widely acknowledged as a significant substrate for autophagy that targets proteins for degradation while also enhancing AD-related pathology through autophagy amplification [23]. Within the context of AD, autophagy may serve to facilitate the elimination of Aβ, ultimately leading to a reduction in its accumulation. The resulting decrease in synaptic damage and related cognitive decline subsequently stemming from Aβ toxicity suggest autophagy as a promising avenue for mitigating the effects of this pathological process [24]. The disruption of autophagy can exacerbate the pathological aspects of AD, according to research [25].The equilibrium of the p62-Keap1-Nrf2 positive feedback loop plays a crucial role in connecting the Nrf2 pathway and autophagy, and may serve as a promising target for treating AD [26].

However, it remains uncertain whether the activation of the p62-Keap1-Nrf2 pathway plays a role in safeguarding against Al-related neurotoxicity and cognitive impairment. As such, the present research endeavors to identify the chemical compounds present in AP extract through UPLC-ESI-qTOF-MS and investigate whether AP can mitigate the effects of Al neurotoxicity and enhance cognitive functions in an alchlor/D-gal induced model and in aluminum maltolate (Al(mal)_3_)-induced PC12 cells. Furthermore, we have evaluated the impact of AP on the p62-Keap1-Nrf2 pathway to gain insight into the potential mechanism behind AP’s effect.

## Methods

### Chemicals

Andrographolide (purity ≥ 98%, B20207), neoandrographolide (purity ≥ 98%, B21275) and deoxyandrographolide (purity ≥ 98%, B21392) were purchased from Yuanye bio-technology Co.,Ltd.(Shanghai, China); Aluminum chloride hexahydrate (AlCl_3,_ 97% purity) and maltol (99% purity) were purchased from Aladdin (Shanghai, China); D-gal(purity 99%) was purchased from Biosharp (Shenzhen, China); Donepezil hydrochloride (98%, D859456) was purchased from Macklin biochemical technology Co., Ltd (Shanghai, China). The preparation method of Al(mal)_3_ referred to our previously published literature [27].

### Preparation of *Andrographis paniculata* ethanol extract

*Andrographis paniculata* (AP, lot number:18,006) were purchased from Huadong Medicine Co., Ltd (Hangzhou, China). The extraction processes and analytical techniques used to acquire and standardize AP have been described in detail here. Ultrasonic extraction with 95% ethanol for 1 h, and the ratio of material to liquid was 1:15. Then the filtrate was spun dry with a vacuum rotary evaporator. Ethanol extract of AP (refer to AP extract in this study) was diluted to different mass concentrations and stored at 4 °C, and it would be filtered through a 0.22 μm micropore filter to use in vitro experiment.

### UPLC-ESI-qTOF-MS analysis of the constituents from *Andrographis paniculata*

AP extract (10.9 mg) was solubilized in methanol (10 mL) under an ultrasound. The sample solution was filtered with a 0.22 μm membrane and transferred into a sample bottle for testing. Andrographolide (10.5 mg), neoandrographolide (10.6 mg) and deoxyandrographolide (10.6 mg) were solubilized in methanol (10 mL) under an ultrasound. UPLC-MS/MS analyses were performed using the UPLC system (Waters) with an ACQUITY UPLC BEH C18 column (100 mm×2.1 mm, 1.7 μm). The column temperature was 40℃, injection volume was 5 µL and the flow rate was 0.35 mL/min. A 0.1% formic acid-water (v: v) solution was used as mobile phase A and acetonitrile as mobile phase B. The gradient elution conditions were set as follows: 0-0.2 min: 10% B; 0.2–15.0 min, 10-40% B; 15.0–22.0 min, 40–85% B; 22.0–24.0 min, 85% B; 24.0-24.5 min, 85 − 10% B; 24.5–28 min, 10% B.All mass spectrometric data were acquired by a Waters Synapt G2 Q-TOF system (Waters Manchester, UK) equipped with an electrospray ionization source that operated in both the positive ion mode (ESI^+^) and negative ion mode (ESI^−^). The mass spectrometric data were collected from m/z 100 to 1,000 in the positive and negative ion modes under the centroid mode. Data acquisition and processing were performed using MassLynx V4.1 software (Waters Corporation).

### Animal treatment

Male ICR mice (18–20 g) were purchased from Zhejiang experimental animal center (license No. SYXK 2019-0011). All the animal experiments were approved by the Institutional Animal Care and Use Committee of Zhejiang Center of Laboratory Animal (Approval No.ZJCLA-IACUC-20,070,022). All animals were acclimatized in the room with humidity of 30–55% and temperature of 22–25℃ for one week. The mice (n = 100) were randomly divided into normal (n = 10) and AlCl_3_/D-gal-treated (n = 90) group. The AlCl_3_/D-gal-treated mice were assigned into 6 experimental groups randomly (n = 15 in each group): [1] AD model control (vehicle-treated); [2] AD + Andro 2 mg/kg/2 days; [3] AD + AP low (200 mg/kg/day); [4] AD + AP middle (400 mg/kg/day); [5] AD + AP high (600 mg/kg/day); [6] AD + donepezil 3 mg/kg/day (reference or positive control group). First, mice except the normal group were given AlCl_3_ orally at 20 mg/kg/d for 4 weeks. The normal group was given equal volume of double distilled water by intragastric administration. Then, mice except the normal group were intraperitoneally injected with D-gal 120 mg/kg/d and given AlCl_3_ orally 20 mg/kg/d for 2 weeks. After that, mice except the normal group were administrated with AlCl_3_/D-gal in the morning. And in the afternoon, mice from treatment group were intraperitoneally injected with andrographolide or donepezil or given AP extract orally for 5 weeks. Normal and AD model group mice were given equal volume of double distilled water by intragastric administration. (volume-matched).

### Morris water maze

The Morris water maze test is a widely-used behavioral task for investigating spatial learning and memory in animals. The test involves placing mice in a circular pool of water with specific visual clues to help them locate a hidden platform. The platform is submerged one centimeter below the water surface and measures ten centimeters in diameter. The pool is divided into four quadrants of equal size. Mice are given four daily acquisition training sessions with 5-min intertrial intervals over four consecutive days. During each session, mice are allowed to locate the platform for 60 s and rest for 5 s upon reaching it. If a mouse fails to locate the platform, it is placed on the platform for a 10-second rest period. After the final acquisition trial, a single 60 s probe trial is conducted to evaluate spatial memory retention. During the trials, the swim latency, path length, swimming speed, and frequency of entry to the target area (during the probe trial) are all recorded using the ANY-maze Video Tracking System (Stoelting, Wood Dale, IL, USA). All collected data are used to evaluate the performance of the water maze task. The escape platform remains in the same location throughout the training trials, while it is moved away during the probe test.

### Sample collection

Subsequent to the behavioral tests, the mice were administered anesthesia and subjected to blood collection before being humanely euthanized. The brain was promptly placed on ice and the hippocampus was meticulously dissected, snap frozen in liquid nitrogen, and securely stored at a temperature of -80℃ for future processing needs.

### Hematoxylin & Eosin (HE) staining

Histopathological studies were performed in the hippocampus. The brain tissues were fixed with 10% buffered formalin at room temperature for 48 h. Then the brain was paraffin-embedded and cut into 4-µm-thick coronal sections. Sections through the hippocampus were deparaffinized, rehydrated, stained with hematoxylin and eosin, and visualized under a light microscope (Olympus,Japan).

### Biochemical assay

The brains were processed into tissue homogenate using a 0.9% saline solution in order to assess protein concentration using a BCA protein assay kit (Beyotime, Shanghai, China). The levels of malondialdehyde (MDA) and activity of superoxide dismutase (SOD) in both brain samples and serum were measured utilizing commercial MDA and SOD kits (Nanjing Jiancheng Bioengineering Institute, China), following the manufacturer’s instructions.

### Cell culture and MTT assay

PC12 cells are a rat adrenal pheochromocytoma cell line, a monoclonal cell line transplanted from rat adrenal medulloblastoma by Greene and Tischler in 1976 [28]. However, PC12 cells differentiate into sympathetic nerve-like cells under the induction of nerve growth factor (NGF), which are close to neurons in terms of morphology, physiological and biochemical functions, such as growing cell protrusions, forming synapse-like structures, and having electrical excitability properties. Furthermore, under the action of NGF, they can synthesize acetylcholine and form neurite structures [29]. Additionally, the PC12 cell membrane has IV-methyl-D-aspartic acid (IV-methyl-D-panic acid, NMDA) receptors (NMDARs, as excitatory amino acid receptors in the central nervous system) that regulate synaptic plasticity, memory, and cognitive ability. The weakened nerve conduction function mediated by NMDARs can lead to brain aging, neuroplasticity damage, and cognitive dysfunction. In addition, NMDARs can interact with amyloid β-peptide/amyloid precursor protein and tau protein [30]. Therefore, PC12 cells are generally used as an ideal cellular model to study pathological molecular mechanisms of AD.

The highly differentiated PC12 cells were obtained from the cell center of the Chinese Academy of Medical Sciences, located in Beijing, China. These cells were maintained in Dulbecco’s Modified Eagle’s Medium (DMEM) supplemented with 10% fetal bovine serum (FBS) and 0.1% penicillin/streptomycin under optimal conditions of 37 °C and 5% CO2. An AD cell model was established using Al(mal)_3_, and cell viability was assessed using the MTT assay. The cells were seeded in a 96-well plate at a density of 100 µL/well and categorized into normal, Al(mal)_3_ treatment and Al(mal)_3_ + AP co-treatment groups for 24 h. Then, MTT solution (20 µL of 5 mg/mL) was added, and after 4 h of incubation, the culture medium was removed, and DMSO (150 µL/well) was added. The absorbance was determined at 570 nm using a Cytation 1 imaging reader (BioTek, USA).

### MRFP-eGFP-LC3 transfection

Cells were inoculated into 6-well plates at a density of 2 × 10^5^ cells/mL, and transfection began when the growth fusion approached 70–90%. Add 3.75 µL Lipofectamine™ 3000 (Invitrogen, USA) into 125µL FBS-free DMEM medium and mix well. Add 2.5 µg LC3 plasmid into 125µL FBS-free DMEM medium, then add 5µL P3000TM reagent and mix thoroughly. Then the two tubes were blended and hatched at room temperature for 10 min. The above mixture was evenly dropped into the cell culture plate and placed in an incubator for 48 h. PtfLC3 was a gift from Tamotsu Yoshimori (Addgene plasmid#21,074; http://n2t.net/addgene: 21,074; RRID: Addgene_21074)). After 24 h with AP extract or Al(mal)_3_ treatment, the changes of red and green LC3 bright spots in the cells were observed under an inverted fluorescence microscope. The ptfLC3 could express mRFP-EGFP-LC3 fusion protein, in which mRFP emits red fluorescence and eGFP emits green fluorescence. The bright yellow spots represent autophagosomes and the bright red spots represent autophagolysosomes in the red and green merge images.

### Transient gene silencing by small interfering RNAs (siRNAs)

Cells were inoculated into a 6-well plate for 24 h prior to transfection. p62 siRNA (20 µM) was transfected into PC12 cells using Lipofectamine^™^ 3000 transfection reagent (Invitrogen USA) according to the manufacturer’s instructions. The p62 siRNA duplexes were synthesized, and sequences were as follows: 5′-UAUCAGUUGUACUAAUCCCUU-3′ (sense), 5′- GGGAUUAGUACAACUGAUAGU-3′ (antisense). After 12 h, cells were grouped and were subjected to AP extract or Al(mal)_3_.

### Quantitative PCR (RT-qPCR) analysis

Total RNAs were extracted from cells by using the RNAprep pure cell/bacteria kit (DP430, Tiangen, China). Then reverse transcribed into cDNA by Fasting gDNA Dispelling RT SuperMix (KR116, Tiangen, China). The RT-qPCR analysis was conducted using ABI 7500 fast system (Applied Biosystems, USA) with SuperReal PreMix Color (SYBR Green, FP205). The primers were synthetized from Sangon Biotech (Shanghai, China) and shown as follows: β-actin: 5′-GCAGGAGTACGATGAGTCCG-3′ (forward), 5′-ACGCAGCTCAGTAACAGTCC-3′ (reverse); p62: 5′-GTCAATTTCCTGAAGAATGTGGG-3′ (forward), 5′-GAGTTCACCTGTGG ATGGGTC-3′ (reverse); Nrf2: 5′-GCCCTCAGCATGATGGACTT-3′ (forward) and 5′-GTTTGGGAATGTGGGCAACC-3′ (reverse); Keap1: 5′-TGGGTCAAATACGACTGCCC-3′ (forward) and 5′- TGGCTCATATCTCTCCACGC-3′ (reverse). Analysis of melting curve data to identify the specificity of PCR. Relative fold expressions were analyzed using the 2-^ΔΔCt^ method and using β-actin Ct values as the internal reference in each sample.

### Western blot analysis

The samples from hippocampus tissues or cells were lysed and centrifuged, the supernatant was collected and mixed with 5×loading buffer at a volume ratio of 1:4, and denatured at 100℃for 30 min. Each sample was isolated by SDS-PAGE and then transferred onto membranes. The membranes were then blocked with 5% (w/v) fat-free milk for 1 h, followed by incubation overnight at 4 °C with primary antibodies at 1:1000 dilution ratio: Nrf2(AF7623, Beyotime, Shanghai, China); Keap1 (sc-514,914, Santa Cruz); Tau (ab32057, Abcam); phospho-Tau (ab109390, Abcam); p62 (AF5384, Affinity, USA); LC3B (L7543, Sigma); GAPDH (FD0063, Fudebio, China). After cleaning, the membranes were incubated with horseradish peroxidase (HRP)-conjugated secondary antibodies for 1 h. The membranes were washed in the western lighting plus-ECL solutions, and lastly the immunoreactive bands were measured using a Chemiluminescence imaging system (Amersham Imager 800, Cytiva). The related optical density of the digitized image was analyzed by ImageQuant TL 8.1 software.

### Statistical analysis

Statistical analysis was conducted using GraphPad Prism 8.0 statistical software (Graphpad Inc, San Diego, CA, USA). All data were displayed as the mean ± SD. The P-values were calculated using a one-way analysis of variance (ANOVA) or Tukey’s multiple comparisons test. A P-value of < 0.05 was regarded as indicating a statistically meaningful result.

## Results

### Chemical composition of AP ethanol extract

**Fig. 1:**
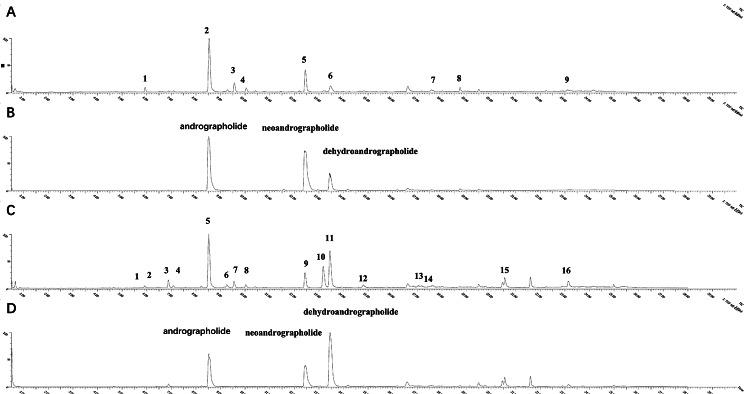
The total ion chromatogram (TIC) of AP extract by UPLC-ESI-qTOF-MS/MS analysis in ESI- (**A**) and ESI+ (**C**) and the TIC of 3 mixed standards by UPLC-ESI-qTOF-MS/MS analysis in ESI- (**B**) and ESI+ (**D**)

The response values of different chemical components in the negative ion mode and positive ion mode are different. Based on the UPLC-ESI-qTOF-MS/MS chromatogram of AP ethanol extract, great segregation was accomplished in 28 min. By comparing the retention time (t_R_), accurate quality and fragmentation patterns that were reported in the literature [12, 31, 32]. 21 chemical compositions were confirmed from ethanol extract of AP **(**Fig. 1), and their results were indicated in Supplementary Tables 1 and 2. Among them, compositions verified by chemical reference standard comparison are indicated by the symbol “▲” in Supplementary Table S1 and Table S2, and the results are indicated in Fig. 1B and Fig. 1D. 21 compounds, including 12.13-dihydroandrographolide, andrographolide, 14-Deoxy-11,12 didehydroandrographolide, 14-Deoxyandrographiside, 2’-Hydroxy-2,4’,6’-trimethoxychalone, neoandrographolide, deoxyandrographolide, dehydroandrographolide, andrographolactone and andrograpanin were identified from AP extract and 18 compounds belong to terpenoids or terpenes. Besides, area normalization analysis at 254 nm was conducted, and the peak area ratio of the three major components (andrographolide, neoandrographolide and dehydroandrographolide) discovered in AP extract consistently surpassed 70%, indicating that the selected compounds were the primary effective ingredients of the ethanolic extract of AP.

### AP ameliorates cognitive impairment in Alchor/D-gal-treated mice

**Fig. 2:**
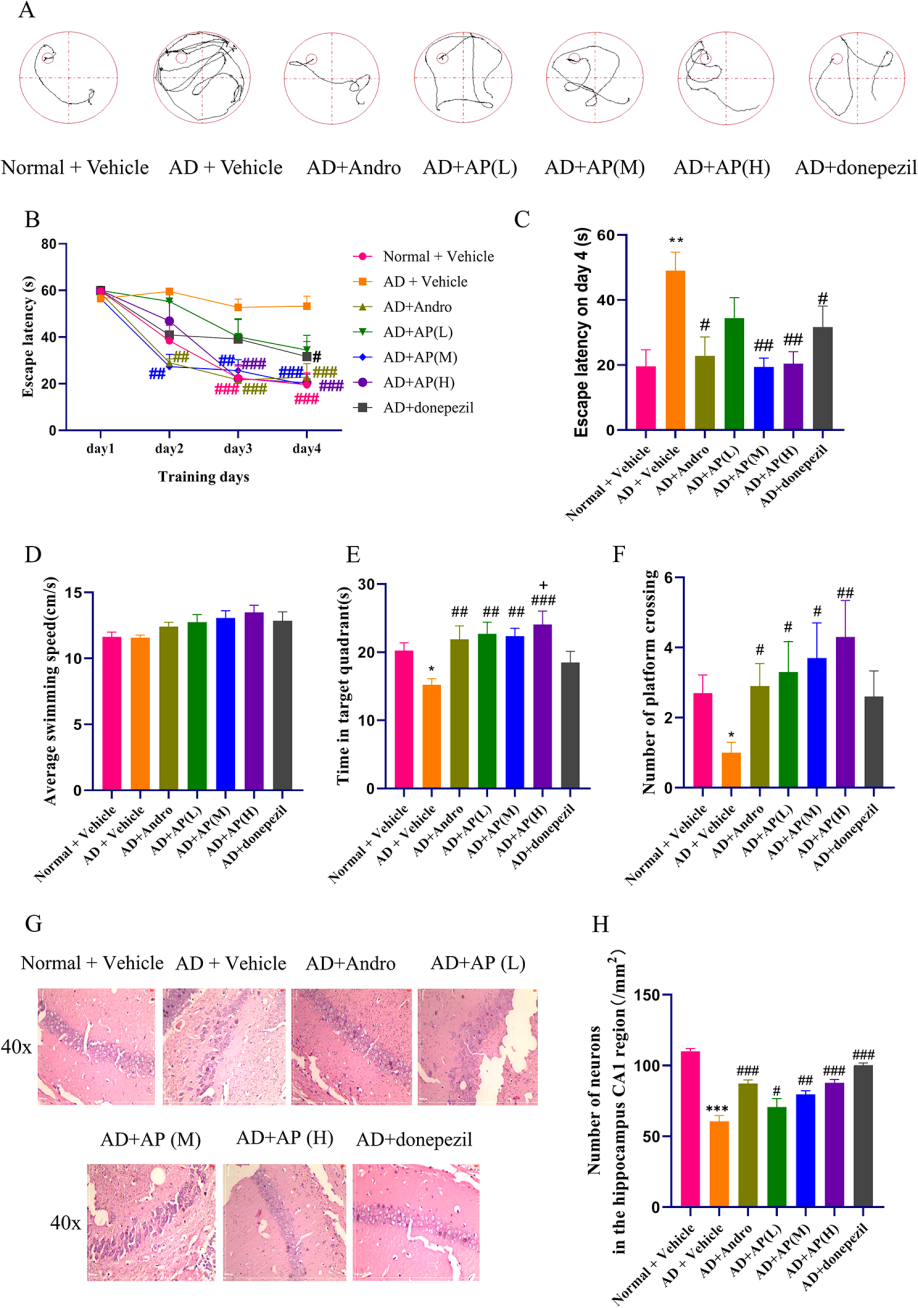
The effect of AP and andrographolide on cognitive functions in alchor/D-gal induced AD mice. MWM test was conducted to assess the cognitive capacity of the mice after treatment with AP and andrographolide. (**A**) Typical swimming paths in the training trials on day 4; (**B**) Line chart of time needed to find the hidden platform (escape latency) from day 1 to day 4 during the training trials; (**C**) Quantitative analysis of escape latency of mice on day 4; (**D**)Average swimming speed in the probe trails; (**E**) Percent of time spent in the target quadrant; (**F**)Number of platform crossing. model group;(**G**) HE staining of the hippocampal CA1 region (original magnifcation, ×400, scale bars = 200 μm); (**H**) Number of neuronal cells in hippocampus CA1. Values are mean ± SD (n = 10). *P < 0.05, **P < 0.01, ***P < 0.001 vs. normal group, ^#^*P* < 0.05, ^##^*P* < 0.01, ^###^*P* < 0.001 vs. model group, ^+^*P* < 0.05 vs. donepezil group

To investigate the neuroprotective role of AP and andrographolide on alchor/D-gal induced AD mice, the MWM task to test spatial learning and memory was implemented. Figure 2 A shows the representative swimming routes of mice in each group during training trails on day 4. The learning curve (mean escape latency) in the acquisition trials is shown in Fig. 2B. The escape latency of model group was significantly longer than the normal group(*P* < 0.01), suggesting slower spatial learning. The defects were partly reversed by AP and andrographolide treatment. The escape latency in andrographolide, AP(M), AP(H) and donepezil groups significantly decreased compared with that in the model group on day 4(Fig. 2C, P < 0.05, *P* < 0.01), there was a downward trend but no difference in AP(L) group. And the escape latency in andrographolide, AP(M) and AP(H) group were less than donepezil group.

Through swimming speeds, number of platform crossings and time spent in the target quadrant to measure, in the probe trial the performance among groups also varied. There was no remarkable distinction in terms of swimming speeds among all groups but the AP treatment group showed a dose-dependent increase in Fig. 2D. Mice in the model group spent less time in the target quadrant (Fig. 2E, P < 0.05), and noticeably decreased the number of platform crossings (Fig. 2F, P < 0.05) compared with that in the normal group. Compared with the model group, the time spent in the target quadrant in AP, andrographolide were significantly increased (*P* < 0.01, *P* < 0.001) and the number of crossing platforms was remarkably elevated (*P* < 0.05, *P* < 0.01). The AP treatment group showed a dose-dependent increase in the number of crossing platforms. The time spent in the target quadrant in AP or andrographolide-treatment were higher than that in donepezil group, and there was significant difference in the AP(H) group(*P* < 0.05). Those results indicated that AP or andrographolide could enhance the cognitive function of alchor/D-gal induced AD mice.

The results of HE staining show the vertebral neurons in the CA1 region of the hippocampus, which is the main cell type of hippocampal neurons. As shown in Fig. 2G, hippocampal neurons of the normal group were densely packed together and the nucleus was centered with clear staining. On the contrary, neurons of the model group were loosely arranged, condensed nuclei, and even disappeared. However, these neural damages were alleviated after AP or andrographolide treatment. As shown in Fig. 2H, Quantitative results showed a consistent trend, but there was no significant difference between AP and andrographolide administration group compared to the model group.

### AP changes the levels of SOD and MDA in serum and brain from Alchor/D-gal-treated mice

**Fig. 3:**
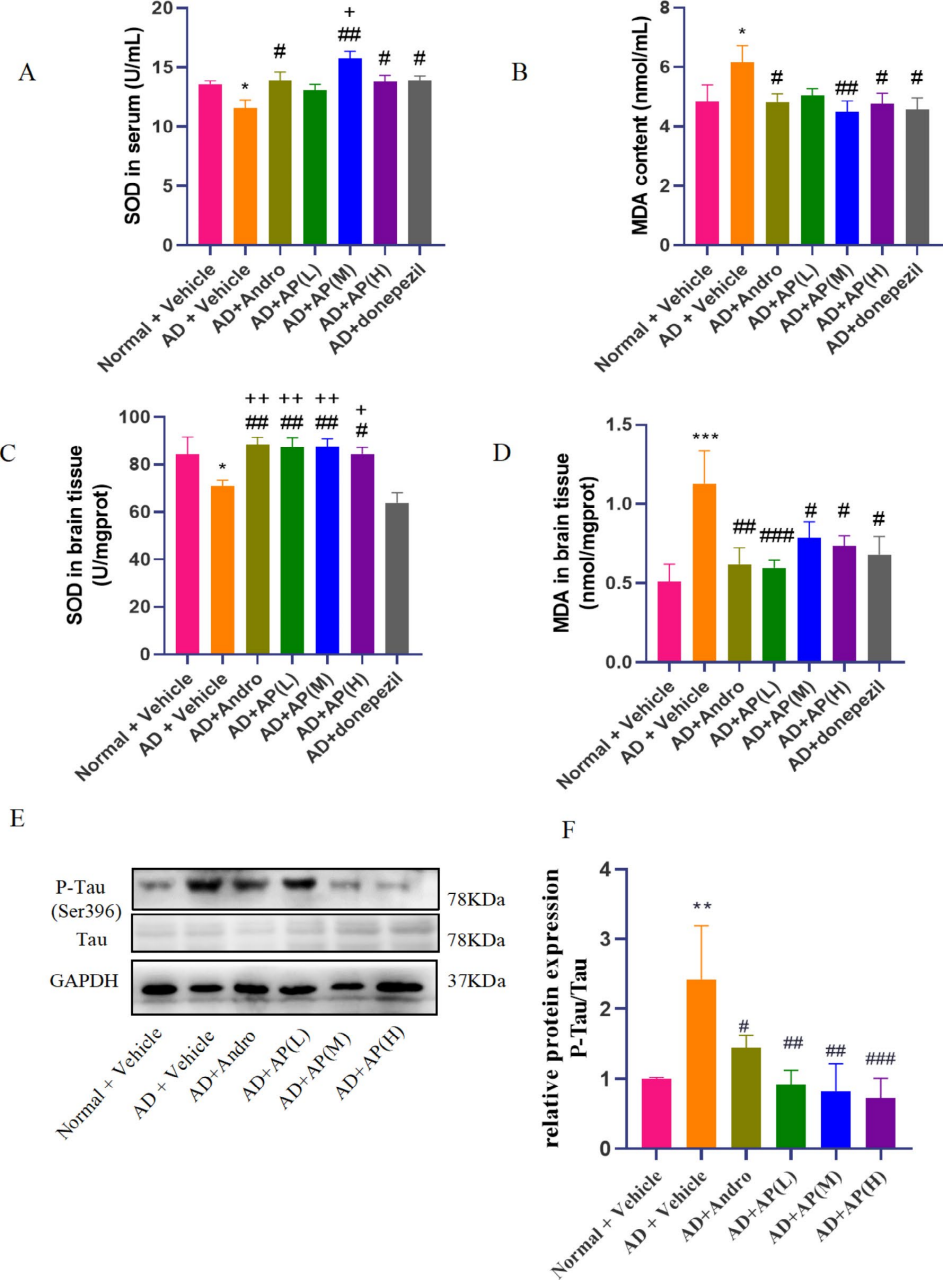
Effect of AP and andrographolide on oxidative stress markers and Tau protein in alchor/D-gal induced AD mice. The SOD activity and MDA content in serum(**A**-**B**) and brain tissue(**C**-**D**) were determined by commercially-available kits. Values are mean ± SD (n = 8–10) (**E**) The expression levels of Tau and P-Tau in the hippocampus were examined by western blot, and GAPDH was served as a loading control. (**F**) Quantitative analysis of P-Tau/Tau protein expression.Values are mean ± SD (n = 6–8). ^*^P < 0.05, ^***^P < 0.001 vs. normal group, ^#^*P* < 0.05, ^##^*P* < 0.01, ^###^*P* < 0.001 vs. model group, ^+^*P* < 0.05, ^++^*P* < 0.05 vs. donepezil group

Oxidative stress is a pathological feature of AD, which can be assessed by the content of MDA and SOD activity. Compared with the normal group, mice in the model group had a lower SOD activity (*P* < 0.05, Fig. 3A) and a higher MDA content (*P* < 0.05, Fig. 3B) in serum. After treatment with AP (400 and 600 mg/kg), andrographolide or donepezil, the unusual levels of SOD and MDA in serum of model mice were successfully saved (*P* < 0.05, *P* < 0.01). The SOD activity from mice serum in the AP middle (400 mg/kg) group in particular increased from 11.57 to 15.77 U/mL over other groups, and also was significantly higher than donepezil group(*P* < 0.05).

In brain tissue, mice in the model group had a lower SOD activity (*P* < 0.05, Fig. 3C) and a higher MDA content (*P* < 0.001, Fig. 3D) compared with the normal group. The SOD activity from mice brain in the AP (200, 400 and 600 mg/kg) and andrographolide groups increased compared with model group or donepezil group (*P* < 0.05, *P* < 0.01). The MDA content from mice brain in the AP (200, 400 and 600 mg/kg), andrographolide or donepezil groups decreased compared with model group (*P* < 0.05, *P* < 0.01, *P* < 0.001). These results indicate that AP and andrographolide have better antioxidant stress ability in Alchor/D-gal-treated mice.

### AP decreases tau protein in Alchor/D-gal-treated mice

Intracellular hyperphosphorylation of Tau is one of the pathological features of AD. The results of our experiment showed that compared with the normal group, the ratio of p-Tau ser396 to Tau was higher in the model group (*P* < 0.01), while AP at doses of 200, 400 and 600 mg/kg (*P* < 0.01, *P* < 0.001) and andrographolide (*P* < 0.05) could significantly reduce the ratio of p-Tau to Tau compared with the model group (Fig. 3E and F**)**. The above data indicated that AP could reduce the expression of phosphorylated Tau in the brain of model mice, the greater the dose, the greater the effect.

### AP up-regulates the levels of Nrf2, keap1, p62 and LC3B-II proteins in Alchor/D-gal-treated mice

**Fig. 4:**
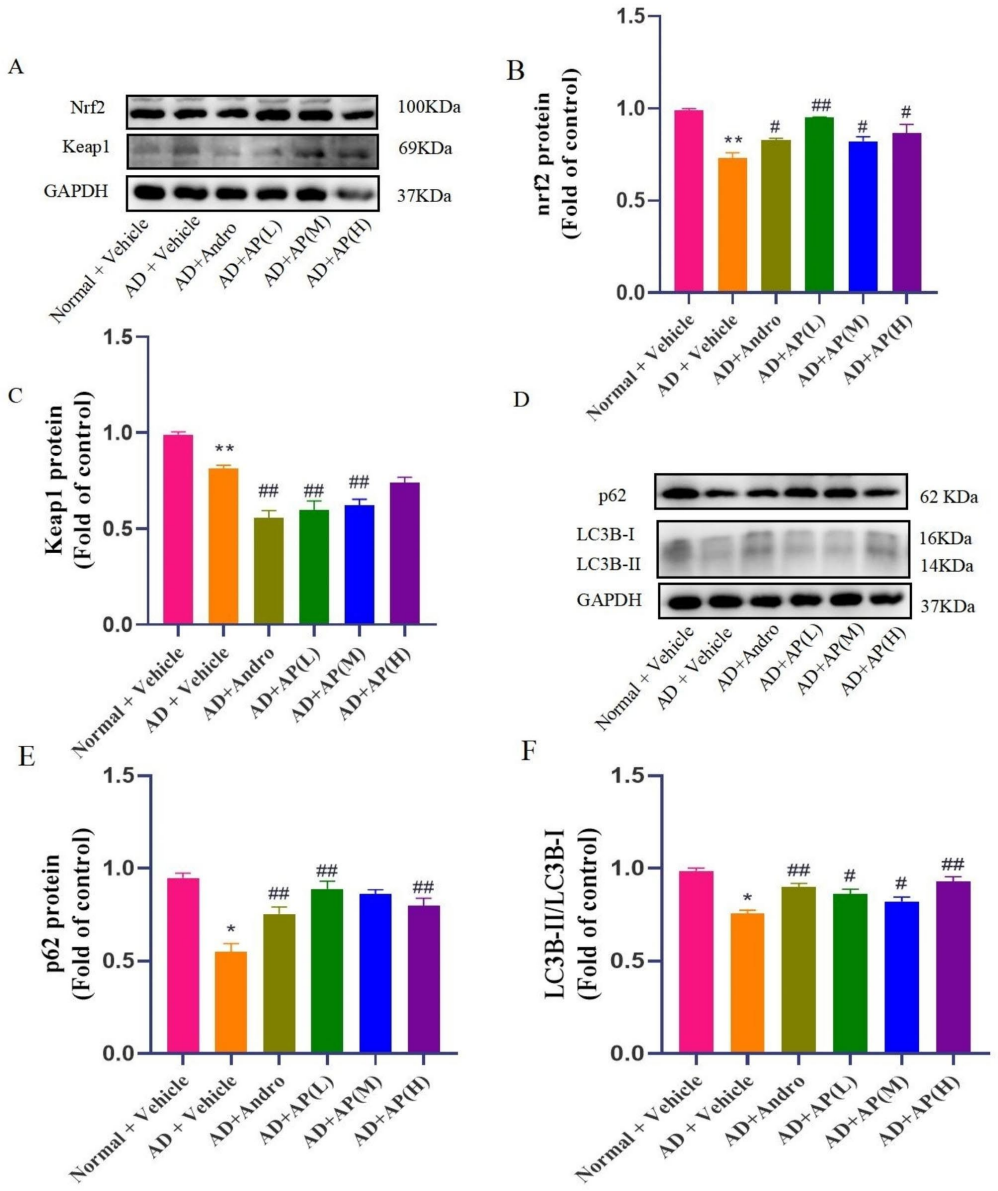
Effect of AP and andrographolide on Nrf2, Keap1, p62 and LC3B-II protein expression in the hippocampus of alchor/D-gal induced AD mice. The expression levels of Nrf2, Keap1 (**A**), p62 and LC3B-II (**D**) protein were examined by western blot, and GAPDH was served as loading control; Quantitative analysis of Nrf2 (**B**), Keap1 (**C**), p62(**E**) and LC3B-II (**F**) protein expression. Values are mean ± SD (n = 6–8). ^*^P < 0.05, ^**^P < 0.01 versus the normal, ^#^*P* < 0.05, ^##^*P* < 0.01 versus model group

As shown in Fig. 4A, compared with the normal group, mice in the model group appeared a decrease in expression of Nrf2 protein(*P* < 0.05). Compared with the model group, andrographolide and AP can significantly increase the expression of Nrf2(*P* < 0.05, Fig. 4B). As for the expression of Keap1, which plays a primary role in Nrf2 degradation, mice in the model group appeared a decrease in expression of Keap1 protein compared with the normal group (*P* < 0.05). Compared with the model group, andrographolide and AP (200 and 400 mg/kg) significantly downregulated expression of Keap1 (*P* < 0.05) while AP 600 mg/kg had no significant differences (Fig. 4C**)**. As shown in Fig. 4D, compared with the normal group, the expression of p62 and LC3B-II protein were downregulated in the model group(*P* < 0.05). Compared with the model group, andrographolide and AP upregulated the expression of p62 and LC3B-II protein (*P* < 0.05, Fig. 4E-F). These data suggested that the p62-Keap1-Nrf2 positive feedback loop may be an important mechanism involved in treating senile dementia by AP or andrographolide.

### AP inhibits the cell viability decline and induces autophagosome in Al(mal)_3_-treated cells

**Fig. 5:**
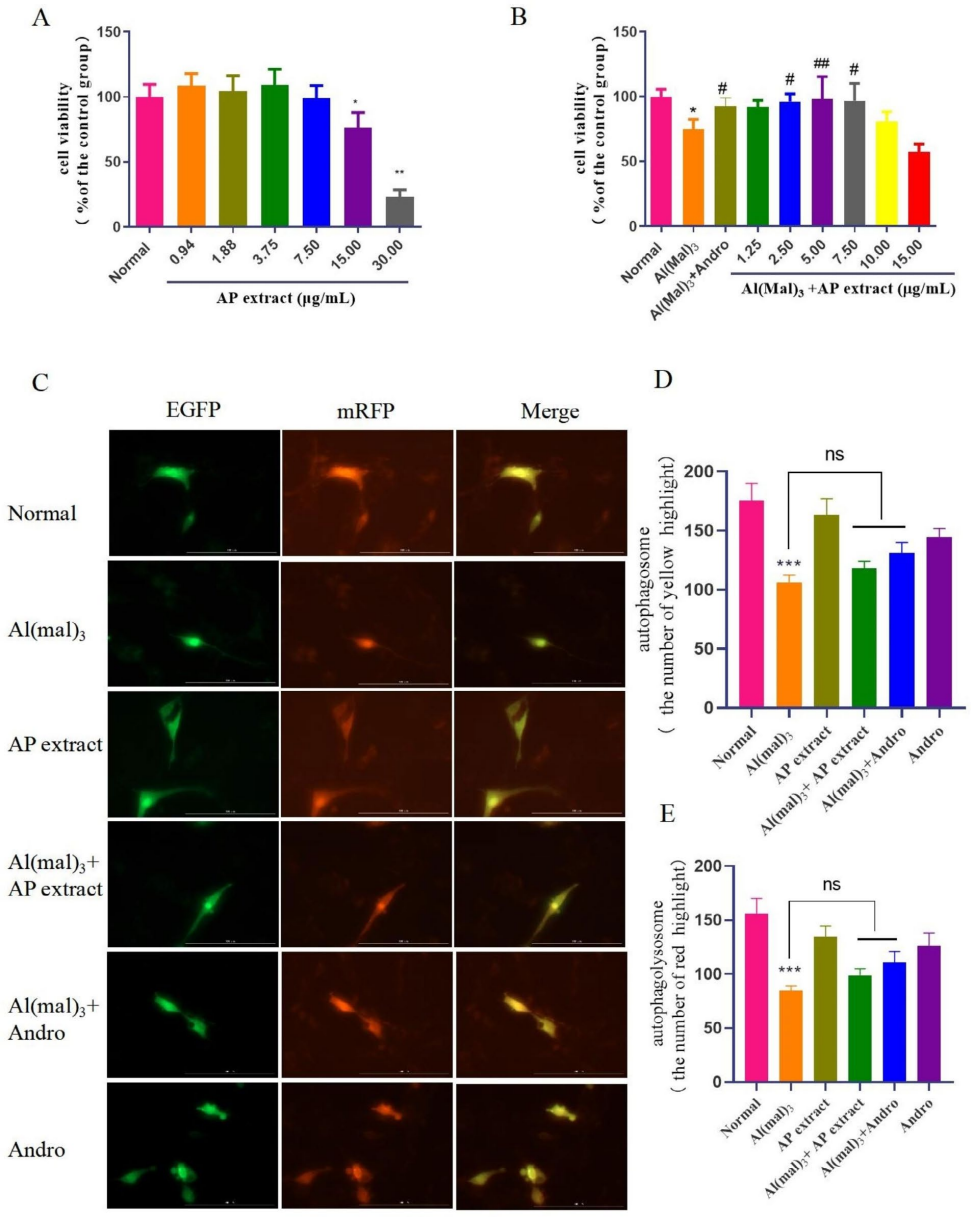
Effect of AP and andrographolide on cell viability and LC3 proteins expression in the AD cell model. (**A**)PC12 Cells were treated with a range of AP concentrations (0.94,1.88,3.75,7.50,15.00 and 30.00 µg/mL) for 24 h; (**B**)Cells were co-treated with 700 µM Al(mal)_3_ and various concentrations of AP (1.25,2.50,5.00,7.50.10.00 and 15.00 µg/mL) or 10 µM Andro for 24 h. Cell viability was measured by MTT assay. (**C**)After transfection with ptfLC3, PC12 cells were incubated with 700 µM Al(mal)_3_ and 5.00 µg /mL AP or 10 µM Andro for 24 h. The images of LC3 protein expression were recorded by a fluorescence microscope. (**D**, **E**)The number of autophagosome (yellow) and autophagolysosome (red) dots per cell were quantified. Values are mean ± SD (n = 3). *P < 0.05, **P < 0.01 ,***P < 0.001versus the normal, ^#^*P* < 0.05, ^##^*P* < 0.01 versus Al(mal)_3_ group

Firstly, we investigated the impacts of various concentrations of AP extract on normal cell viability in PC12 cells. The results indicated that when the concentration reached 15.00 µg/mL, cell growth was significantly inhibited (*P* < 0.05, Fig. 5A). Then the AD cell model was established by Al(mal)_3_ at a concentration of 700 µM. AP extract at doses of 2.50, 5.00 and 7.50 µg/mL and andrographolide (10 µM) can significantly inhibit the cell viability decline caused by Al(mal)_3_(*P* < 0.05, *P* < 0.01, Fig. 5B). 5.00 µg/mL of AP increased Al(mal)_3_-induced cell viability from 74.8 to 98.3%, demonstrating preliminarily the protective effect of AP on the cytotoxicity induced by aluminum.

Then we analyzed the autophagosome maturation using a pH-sensitive tandem fluorescence-labeled autophagy reporter mRFP-EGFP-LC3. MRFP-EGFP-LC3 can discriminate between autophagosome (mRFP+/EGFP+) and autophagolysosome (mRFP+/EGFP-). LC3 carries the red fluorescence of mRFP and the green fluorescence of eGFP. It can concentrate on the autophagy precursor and autophagosome membrane in large quantities, making them appear as bright yellow spots in the red-green composite image. In 700 µM Al(mal)_3_-induced cells transfected with LC3 plasmid (Fig**.**5C), the number of yellow and red bright spots decreased compared with the normal group. That is, the autophagosome production and autophagolysosome decreased, and autophagy was inhibited. When 5.00 µg/mL AP extract or andrographolide (10 µM) was added, the number of yellow and red spots increased. As shown in Fig. 5D-E, Quantitative charts showed consistent trends, but the AP and andrographolide administration groups did not show significant differences compared to the model group. The results showed that the autophagy activity was enhanced, consistent with the western blot detection results of LC3B-II protein in mice.

### Silencing p62 reverses the upregulation expression of Nrf2 and downregulation of Keap1 and tau proteins induced by AP in Al(mal)_3_-treated cells

**Fig. 6:**
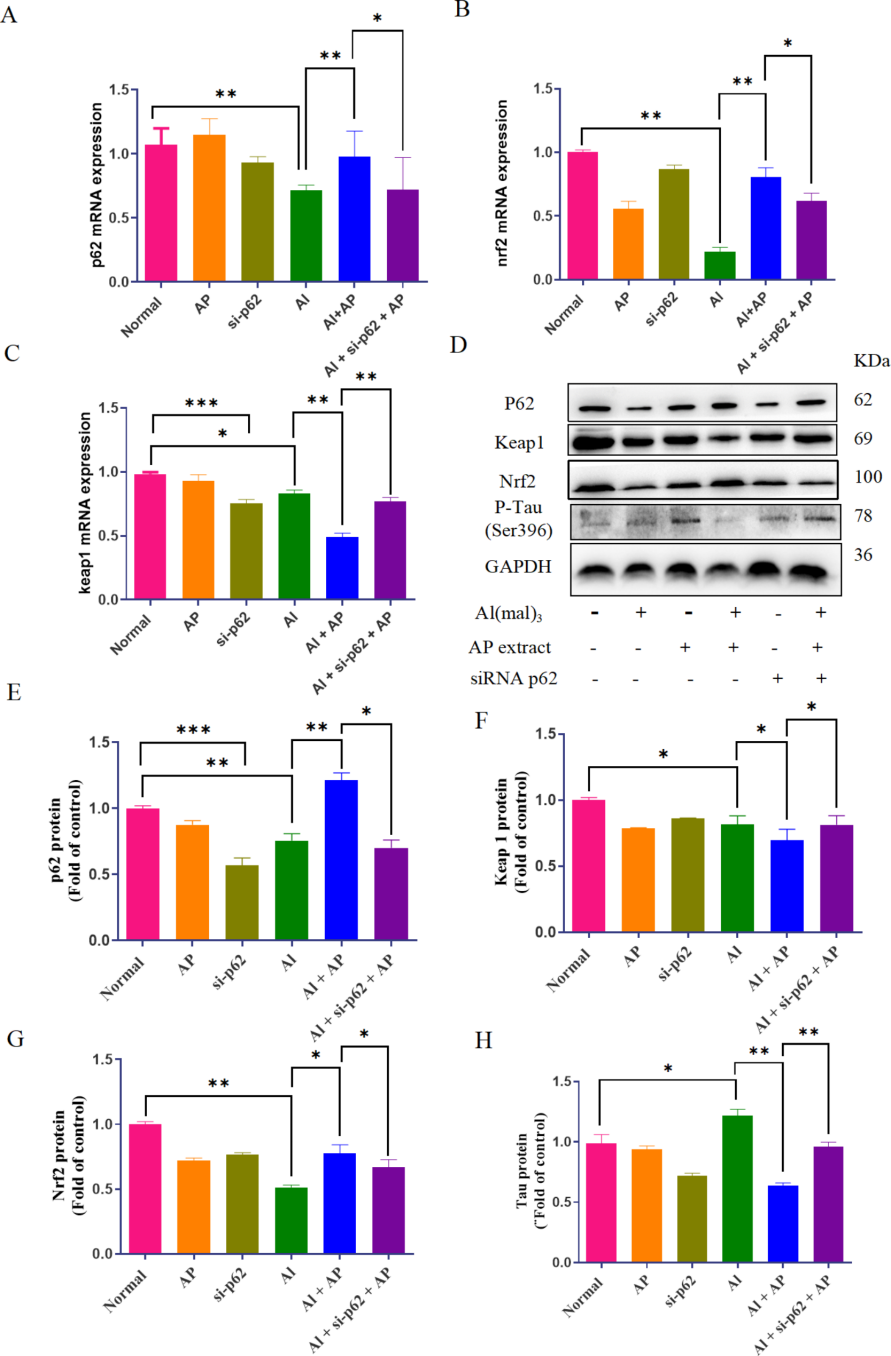
Effect of siRNA p62 on Nrf2, Keap1, p62 and Tau mRNA and proteins expression in the AP-treated AD cell model. After transfection with p62 siRNA, PC12 cells were incubated with 700 µM Al(mal)_3_ and 5.00 µg /mL AP for 24 h. The levels of p62(**A**), Nrf2(**B**) and Keap1 (**C**) mRNA were analysed using RT-qPCR. (**D**) The expression of p62, keap1, Nrf2 and Tau were measured by western blotting, and GAPDH was served as a loading control. Quantitative analysis of p62 (**E**), Keap1 (**F**), Nrf2(G) and Tau (H) protein expression. Values are mean ± SD (n = 3). ^*^P < 0.05, ^**^P < 0.01, ^***^P < 0.001

To further detect the molecular mechanism(s) in which AP modulated PC12 cells, p62 was silenced with small interfering RNA to observe the protein expression changes of keap1 and Nrf2. As shown in Fig. 6A-C, compared with the normal group, the levels of p62, Keap1 and Nrf2 mRNA in Al(mal)_3_-treated cells were decreased (*P* < 0.05, *P* < 0.01). compared with the Al(mal)_3_ group, the levels of p62 and Nrf2 mRNA were upregulated and keap1 mRNA were downregulated in the AP + Al(mal)_3_ group (*P* < 0.05, *P* < 0.01). compared with the AP + Al(mal)_3_ group, the level of Nrf2 mRNA was decreased and keap1 mRNA was increased in siRNA p62 + AP + Al(mal)_3_ group.

The results of this protein level were also consistent in this trial. The expression of p62, Keap1 and Nrf2 proteins in Al(mal)_3_-treated cells were decreased and Tau protein was increased compared with that in the normal (*P* < 0.05, *P* < 0.01).AP treatment strengthened p62 and Nrf2 proteins, and reduced keap1 and p-Tau proteins(*P* < 0.05, *P* < 0.01). Silencing p62 reverses the upregulation of Nrf2 and downregulation of Keap1 and Tau proteins induced by AP (*P* < 0.05, *P* < 0.01, Fig. 6D-H), indicating that the p62-Keap1-Nrf2 pathway was involved in the anti-cytotoxic process of AP.

## Discussion

As per the current study, AP contains significant chemical compounds belonging to the classes of diterpenoid lactones and flavonoids. The potency of their therapeutic effects is correlated with their concentration in the plant. Additionally, the concentration of the solvents utilized during the extraction process plays a crucial role in determining the number of compounds obtained [33]. The current study has successfully identified 18 terpenoids or terpenes, namely andrographolide, neoandrographolide, dehydroandrographolide, deoxyandrographolide, andrographolactone, 14-deoxy-11,12 didehydroandrographolide, 14-deoxyandrographiside, andrographiside, and andrograpanin, within the ethanol extract of AP by utilizing UPLC-ESI-QTOF-MS. The compound andrographolide has shown promising prophylactic and/or therapeutic effects in AD through various mechanisms which include anti-oxidation and anti-neuroinflammation [34]. These active constituents hold considerable potential to explain the diverse biological activities of AP.

According to literatures [15, 16], the intragastric administration of AP extracts in rats had a dose range of 50–500 mg/kg, whereas the same range for mice was 80–800 mg/kg. For our study, low, medium and high dose groups were set up to determine whether AP extracts exert neuro-protective roles or in dose-dependent, so we selected doses of 200, 400, and 600 mg/kg for AP extracts and 2 mg/kg for andrographolide, based on relevant literature [35]. At the same time, we chose Donepezil as our reference group for the following reasons: First, The FDA approved acetylcholinesterase inhibitors Donepezil, providing AD symptomatic relief [36]. Donepezil is a recognized positive drug for relieving AD in the market, and setting Donepezil reference group can be compared with andro and AP extract group to observe their therapeutic degree for AD. The objective of using andrographolide in this study was twofold- to evaluate its impact on the Al-induced model in mice and compare its efficacy and mechanism with that of AP extract. The Alchor/D-gal induced AD model has been extensively described in literature [37, 38], and effectively simulates AD symptoms. In order to evaluate spatial memory, the MWM test is commonly implemented. A reduction in latency time during repeated trials and an increase in time spent in the target quadrant during the probe task is indicative of intact learning and memory function. Our study found that alchor/D-gal-treated mice demonstrated impairments in learning and memory. Conversely, treatment with AP or andrographolide resulted in a decreased latency time in repeated trials, an increased time spent in the target quadrant and an increase in the number of platform crossings during the probe task, with no significant differences between the two treatments. Meanwhile the data also showed that AP has a better effect on improving learning and memory under high dose (600 mg/kg). These outcomes preliminarily demonstrate that AP or andrographolide effectively improve cognitive learning in AD model mice induced by alchor/D-gal.

Impaired redox balance and elevated levels of phosphorylated tau protein are common pathologic features of AD, which also appears in the model of alchor/D-gal. Notably, AP (at doses of 200, 400, and 600 mg/kg) and andrographolide administration yielded a marked decrease in oxidative stress levels assessed via the MDA marker in both the blood and brain tissues, while concomitantly increasing SOD activity, to some extent, the effect was better than that of donepezil positive group. Preliminary findings suggest that the administration of AP or andrographolide may be efficacious in combating oxidative stress and maintenance of homeostasis in vivo. Further, the administration of AP or andrographolide elicited a significant reduction in the expression of p-Tau protein, and there was no difference observed between the AP and andrographolide group.

Subsequently, we conducted further investigation into the molecular mechanism of AP about the treatment of AD symptoms. Within this context, Nrf2 stands out as a pivotal transcription factor that modulates cellular antioxidant response. A decline in Nrf2 function has been observed in the brains of AD patients; conversely, heightened Nrf2 function presents promising therapeutic potential in various AD models [39]. Nrf2 is regulated by several biomolecules, including Keap1 [40]. Our prior research demonstrated that andrographolide alleviates Al(mal)_3_-induced neurotoxicity through the Keap1-Nrf2 pathways in PC12 cells [27]. The findings of our present study demonstrate that AP extract reduces cytotoxicity induced by Al(mal)_3_, elevates Nrf2 expression, and decreases Keap1 levels in treated PC12 cells. Further, both AP and andrographolide modulate changes in Nrf2 and Keap1 protein expressions brought on by alchor/D-gal in mice. These data serve to confirm that AP regulates the Keap1-Nrf2 pathway both in vitro and in vivo. It should be noted that the expression level of Nrf2 or Keap1 protein was not dose-dependent with the AP extract, possibly because the protein was regulated by various components in the AP extract and maintained a relative equilibrium state.

Moreover, the research indicates that activation of p62-mediated autophagy can effectively reduce AD-like pathology and cognitive decline [23]. In the pathology of AD, dysautophagy in neurons precedes Aβ deposition. By repairing the lysosomal acid deficiency associated with PSEN1, autophagy disorders and other AD-related pathologies in AD models can be improved [41]. During autophagosome formation, cytosolic Atg known as Light chain (LC)3B-I undergoes post-translational modification to become LC3B-II, which then interacts with autophagy adaptor proteins such as p62, NDP52, and optineurin via their LC3-interacting regions [42]. p62 and LC3B-II are recognized as typical autophagy marker proteins. The outcomes of our recent study revealed that the expression of p62 and LC3B-II was decreased in mice induced by alchor/D-gal, indicating the inhibition of p62-dependent autophagy. However, this phenomenon was reversed by AP treatment in alchor/D-gal-induced mice. Furthermore, the transfection of the mRFP-eGFP-LC3 plasmid into PC12 cells showed that AP treatment could enhance autophagy activity and upregulate the expression of p62 protein in Al(mal)_3_-treated cells. These findings suggest that AP may improve the inhibition of p62-mediated autophagy.

P62 is recognized as a cargo receptor of significance, providing modulation of a range of transcription factors, including Nrf2, and facilitating the degradation of select proteins, including Keap1. Its function as a pivotal modulator of the Keap1-Nrf2 pathway through association with Keap1 has been the subject of study in various conditions, such as neuropathic pain [43]. The p62-Keap1-Nrf2 pathway holds promise as a novel treatment mechanism for AD [26]. In our laboratory’s prior research, it was discovered that andrographolide provided protection to PC12 cells against autophagy-related cell death induced by Aβ. This protection was achieved through the activation of the Nrf2-mediated p62 signaling pathway [22]. In this study, the results have demonstrated that the up-regulation of Nrf2 and the down-regulation of Keap1 and AD marker Tau induced by AP were significantly mitigated through the silencing of p62 expression in Al(mal)_3_-induced PC12 cells. These findings suggest that AP may activate the noncanonical p62-mediated Keap1-Nrf2 pathway.

The emerging information on the anti-inflammatory and antioxidant effects caused by the properties in AP and andrographolide seems to explain many mechanisms at the heart of neuroprotective action [44]. In other models such as lipopolysaccharide-induced cognitive deficits [15], the researchers suggest that AP prevents cognitive deficits by inhibiting inflammatory cytokines and mediators of oxidative stress. And in present study, AP may alleviate neurotoxicity and cognitive impairment of aluminum through the activation of the p62-Keap1-Nrf2 pathway.Additionally, p62 was found to control Nrf2 protein stability via autophagy and to form a positive feedback loop with its transcriptional targets. It is hypothesized that a molecule may be present during the AP treatment of AD, that triggers p62 activation and links it to Keap1 and other cargoes for lysosomal degradation. Consequently, further research is necessary to determine the validity of this hypothesis.

## Conclusions

In conclusion, UPLC-ESI-MS/MS analysis has revealed that AP contains significant amounts of andrographolide, neoandrographolide, and deoxyandrographolide. Our study has demonstrated the neuroprotective effects of AP and its active ingredient, andrographolide, against AD model mice induced by alchor/D-gal and cell model induced by Al(mal)_3_. The cognitive function and spatial memory of mice was improved with the administration of AP extract and andrographolide. Additionally, there was a reduction in the production of p-Tau while oxidative stress marker MDA production was reduced and SOD activity increased. Interestingly, the effects of AP extract and andrographolide on AD treatment were not significantly different. Moreover, our results suggest that AP may alleviate neurotoxicity and cognitive impairment of aluminum through the activation of the p62-Keap1-Nrf2 positive feedback loop. Based on these promising findings, AP holds potential as a drug candidate for the treatment of AD. However, this study has certain limitations: First, the study utilized a wide range of doses for AP extract and andrographolide. We did not provide a more detailed dose-response analysis to determine the optimal dosage for the observed effects. This would provide a clearer understanding of the therapeutic range and potential side effects associated with these compounds. Second, while the study suggests that AP and andrographolide may activate the p62-Keap1-Nrf2 pathway, the exact molecular mechanisms underlying this activation remain unclear. Further experiments or in-depth molecular studies are needed to provide a more comprehensive understanding of how these compounds work at the cellular and molecular levels. In the future studies, we will further optimize the protocol or model and further study the molecular mechanism of AP improving AD.

### Electronic supplementary material

Below is the link to the electronic supplementary material.

Supplementary Material 1

Supplementary Material 2

## Data Availability

The datasets used and/or analysed during the current study are available from. the corresponding author on reasonable request.

## Abbreviations

AD: Alzheimer’s disease
AP: Andrographis paniculata
Andro: Andrographolide
Aβ: Amyloid-β
Keap1: Kelch-like ECH-associated protein 1
Nrf2: Nuclear factor E2 related factor 2
MDA: Malondialdehyde
SOD: Superoxide dismutase
TCM: Traditional Chinese medicine
Al(mal)_3_: Aluminum maltolate
AlCl_3_: Aluminum chloride
D-gal: D-galactose
