# Supplementary material for: Ethanol extract of Andrographis paniculata alleviates aluminum-induced neurotoxicity and cognitive impairment through regulating the p62-keap1-Nrf2 pathway
Source: BMC Complement Med Ther. 2023 Dec 6;23:441. doi: 10.1186/s12906-023-04290-4 (PMC10698961; doi:10.1186/s12906-023-04290-4)
Supplement: Supplementary file 1 — Supplementary Material 1 [file 12906_2023_4290_MOESM1_ESM.pdf]

Supplementary Materials

Table S1 Characterization of the chemical constituents in AP extract by UPLC-ESI-qTOF-MS/MS (ESI-).

No.	t_R (min)	Formula	Fragment ion *m/z*	ppm	Identity	Class
1	5.93	$C_{28}H_{46}O_{11}$	557.2688[M-H]$^+$ 381.1988[M-H- $C_5H_6O_2$-CO_2-2CH_3]$^+$	-0.7	Dihydroxyl dimethyl 19-[(-D-glucopyranosyl)oxy]-19-oxo-ent-labda-8(17),13-dien-16,15-olide	Diterpenoids
2 ▲	8.54	$C_{20}H_{30}O_5$	349.2012[M-H]$^+$ 395.2087 [M-H+CO+H_2O]$^+$, 377.1990 [M-H+CO]$^+$,331.1896[M-H-H_2O]$^+$, 287.2001[M-H -2OCH_3]$^+$	-0.9	andrographolide	Diterpenes
3	9.56	$C_{27}H_{42}O_{11}$	541.2638[M-H]$^+$ 495.2601[M-H -CO-H_2O]$^+$	-2.0	Methyl methoxyl 14-deoxyandrographiside	Diterpenoids
4	10.04	$C_{26}H_{38}O_9$	493.5220[M-H]$^+$ 539.2584[M-H+CH_3+OCH_3]$^+$	-2.2	14-Deoxy-11,12-Didehydro andrographiside	Diterpenoids
5 ▲	12.44	$C_{26}H_{40}O_8$	479.2969[M-H]$^+$ 525.2878[M-H+CH_3+OCH3]$^+$	-3.5	neoandrographolide	Diterpenoids
6 ▲	13.48	$C_{20}H_{28}O_4$	331.1906[M-H]$^+$ 476.1019[M-H+3CH_3]$^+$,221.1184[M-H-3CH_3-2OH-OCH_3]$^+$	-0.9	dehydroandrographolide	Diterpenes
7	17.58	$C_{17}H_{26}O_4$	293.1760[M-H]$^+$	2.4	3,19-Dihydroxy-14,15,16-trinor-ent-labda-8(17),11-diene-13-oic acid	Diterpenoids
8	18.74	$C_{15}H_{18}O_5$	277.1804[M-H]$^+$	-1.1	Paniculide C	Meroterpenoids
9	23.11	$C_{15}H_{20}O_5$	279.2322[M-H]$^+$ 243.8998[M-H-2H_2O]$^+$	-2.5	Paniculide B	Sesquiterpenoids

Table S2 Characterization of the chemical constituents in AP extract by UPLC-ESI-qTOF-MS/MS (ESI+).

No.	t_R (min)	Formula	Fragment ion *m/z*	ppm	Identity	Class

1	5.93	$C_{20}H_{28}O_4$	333.2060[M+H]$^+$ 315.1978[M+H-H_2O]$^+$,297.1856[M+H-2H_2O]$^+$, 285.1682[M+H--H_2O-2CH_3]$^+$, 257.1516[M+H--H_2O-2CH_3-CO]$^+$	-1.8	3-[2-[6-hydroxy-5-(hydroxymethyl)-5,8a-dimethyl-2-methylene-decalin-1-yl]ethylidene]furan-2-one	Diterpenoids
2	6.42	$C_{20}H_{32}O_5$	352.2344[M+H]$^+$ 335.2272 [M+H-H_2O]$^+$,317.2022 [M+H-2H_2O]$^+$,299.2036[M+H-3H_2O]$^+$, 287.2034[M+H-2H_2O-2CH_3]$^+$	4.5	12.13-dihydroandrographolide	Diterpenoids
3	6.88	$C_{20}H_{30}O_6$	367.2166[M+H]$^+$, 679.5128[2M+H-3H_2O]$^+$, 340.2601[M-C_2H_2]$^+$,276.7272[M-C_2H_2-2OH-2CH_3]$^+$,	0.3	(3s,4s)-3-{2-[(1s,4ar,5s,6s,8as)-6-hydroxy-5-(hydroxymethyl)-5,8a-dimethyl-2-methylidene-hexahydro-1h-naphthalen-1-yl] ethenyl}-3,4-dihydroxyoxolan-2-one	Diterpenoids
4	7.08	$C_{20}H_{28}O_4$	333.2082[M+H]$^+$, 315.1974[M+H-H_2O]$^+$, 303.1903[M+H-2CH_3]$^+$, 297.1859[M+H-2H_2O]$^+$, 285.1862[M+H-H_2O-2CH_3]$^+$, 279.0962[M+H-3H_2O]$^+$,275.1638[M+H-2CH_3-CO]$^+$,257.1537[M+H-2CH_3-CO-H_2O]$^+$	4.8	3-{2-[6-hydroxy-5-(hydroxymethyl)-5,8a-dimethyl-2-methylidene-hexahydro-1h-naphthalen-1-yl]ethenyl}-5h-furan-2-one	Diterpenoids
5 ▲	8.53	$C_{20}H_{30}O_5$	351.2188[M+H]$^+$,701.4259[2M+H]$^+$ 333.2074[M+H-H_2O]$^+$, 315.1969[M+H-2H_2O]$^+$,297.1861[M+H-3H_2O]$^+$, 285.1868[M+H-2H_2O-2CH_3]$^+$, 257.1550[M+H-2H_2O-2CH_3-CO]$^+$	4.8	andrographolide	Diterpenes
6	9.27	$C_{20}H_{28}O_4$	333.2073[M+H]$^+$ 315.1955[M+H-H_2O]$^+$, 303.1974[M+H-2CH_3]$^+$,297.1849[M+H-2H_2O]$^+$, 289.2053[M+H-CO_2]$^+$,285.1848[M+H-2CH_3-H_2O]$^+$, 271.1562[M+H-CO_2-H_2O]$^+$, 257.1519[M+H-2CH_3-H_2O-CO]$^+$	2.1	14-Deoxy-11,12 didehydroandrographolide	Diterpenes

7	9.55	$C_{26}H_{40}O_9$	497.2691[M+H]$^+$,993.5446[2M +H]$^+$, 317.2126 [M+H-Glu-H_2O]$^+$, 299.2017 [M+H-Glu-2H_2O]$^+$,287.2811 [M+H-Glu-H_2O-2CH_3]$^+$, 259.1709 [M+H-Glu-H_2O-2CH_3-CO]$^+$	-0.2	14-Deoxyandrographiside	Diterpenoids
8	10.02	$C_{18}H_{18}O_5$	315.1969[M+H]+ 297.1854[M+H-H_2O]$^+$,285.1868[M+H-CH_3]$^+$,257.1557[M+H-CO]$^+$	1.6	2'-Hydroxy-2,4',6'-trimethoxychalone	Flavones
9 ▲	12.43	$C_{26}H_{40}O_8$	481.2789[M+H]$^+$,961.5594[2M +H]$^{+,}$ 319.2278[M+H-Glc]$^+$,301.2174[M+H-Glc-H_2O]$^+$,289.2156[M+H-Glc-2CH_3]$^+$	-2.5	neoandrographolide	Diterpenoids
10	13.18	$C_{20}H_{30}O_4$	335.2219[M+H]$^+$,669.4379[2M +H]$^+$,317.2138[M+H-H_2O]$^+$,299.2025[M+H-2H_2O]$^+$, 287.2024[M+H-H_2O-2CH_3]$^+$,259.1707[M+H-H_2O-2CH_3-CO]$^+$	-3.0	deoxyandrographolide	Diterpenoids
11 ▲	13.44	$C_{20}H_{28}O_4$	333.2050[M+H]$^+$ 665.4041[2M+H]$^+$ 315.1969[M+H-H_2O]$^+$,297.1861[M+H-2H_2O]$^+$,285.1868[M+H-H_2O-2CH_3]$^+$,257.1550[M+H-H_2O-2CH_3-CO]$^+$	-3.3	dehydroandrographolide	Diterpenes
12	14.80	$C_{20}H_{24}O_2$	297.1855[M+H]$^+$ 279.1765[M+H-H_2O]$^+$,253.3033[M+H-CO_2]$^+$, 197.1328[M+H-CO_2-C_2H_2-2CH_3]$^+$	2.0	Andrographolactone	Diterpenoids
13	17.04	$C_{20}H_{30}O_3$	319.2262[M+H]$^+$,301.2155[M+ H-H_2O]$^+$, 289.2169[M+H-2CH_3]$^+$,179.0747[M+H-5CH_3-2OH-OCH_3]$^+$	-3.4	Andrograpanin	Diterpenes
14	17.60	$C_{17}H_{26}O_4$	295.1921[M+H]$^+$, 251.1280[M+H-CO_2]$^+$	4.1	3,19-Dihydroxy-14,15,16-trinor-ent-labda-8(17),11-diene-13-oic acid	Diterpenoids
15	20.55	$C_9H_8O_2$	149.0239[M+H]$^+$, 279.1588[2M+H-H_2O]$^+$	3.4	Cinnamic acid	Phenolic acids

16	23.13	$C_{18}H_{16}O_5$	313.0745$[M+H]^+$, 524.3815$[2M+H-C_5H_5O_2]^+$, 239.2437$[M+H-CO_2-2CH_3]^+$	0.6	5,7-dimethoxy-2-(2-methoxyphenyl)chromen-4-one	Flavones
