# Supplementary material for: Ethanol extract of Andrographis paniculata alleviates aluminum-induced neurotoxicity and cognitive impairment through regulating the p62-keap1-Nrf2 pathway
Source: BMC Complement Med Ther. 2023 Dec 6;23:441. doi: 10.1186/s12906-023-04290-4 (PMC10698961; doi:10.1186/s12906-023-04290-4)

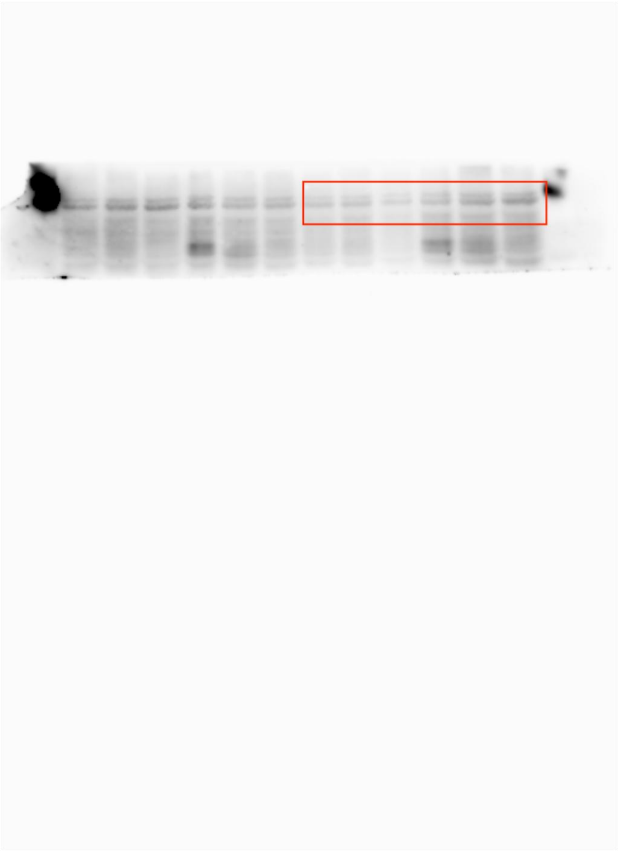

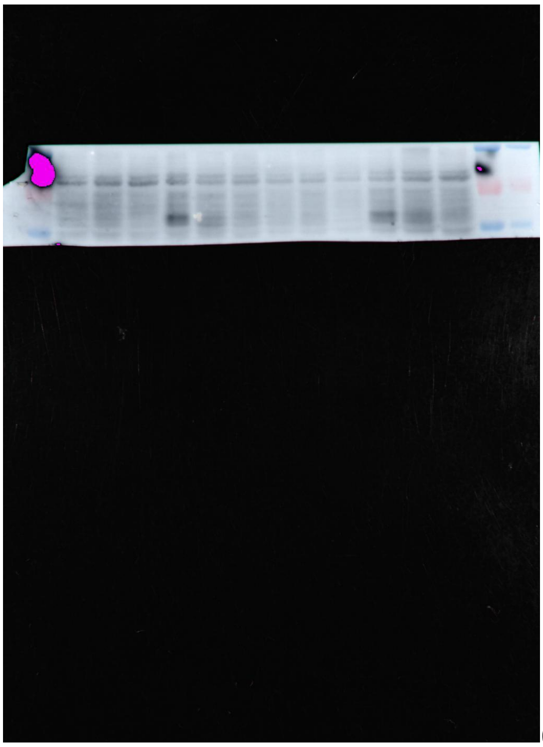

(Taking this picture with boundary marks as an example, we cut through marker to separate the target strip and the internal reference strip, which will not be shown below)

Fig.S1 tau-full unedited gel for Figure 3

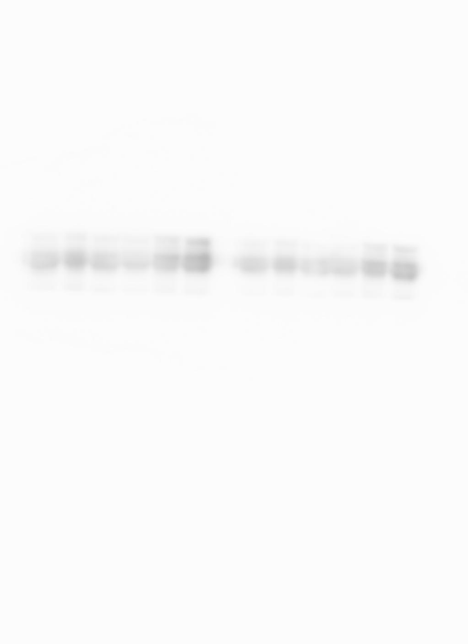

Tau Repeat one:

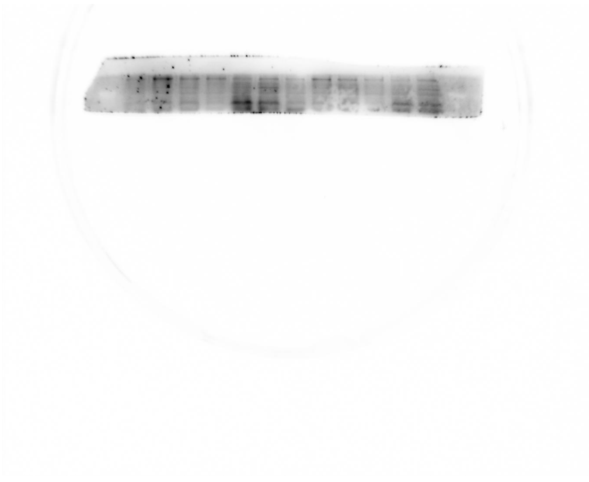

Tau Repeat two:

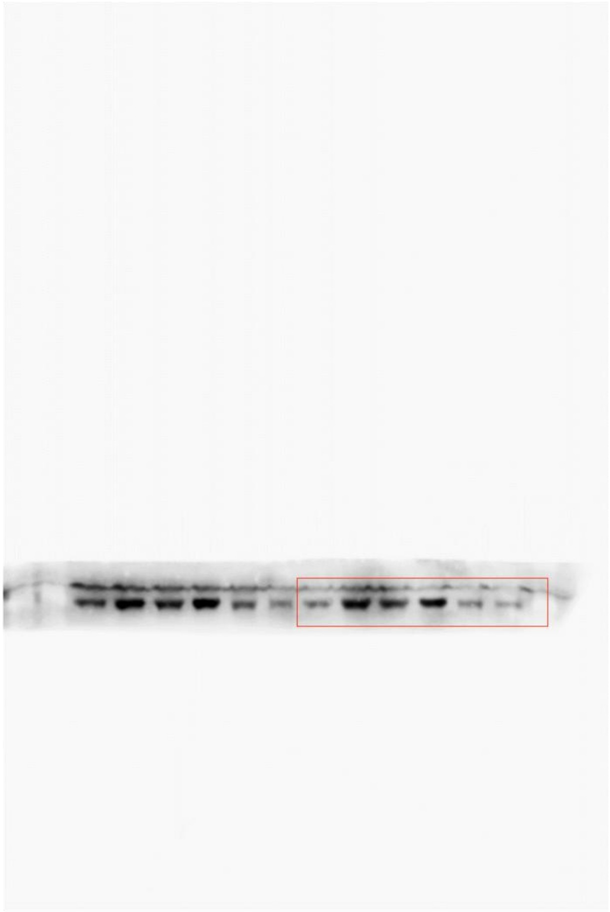

Fig.S2 p-tau-full unedited gel for Figure 3

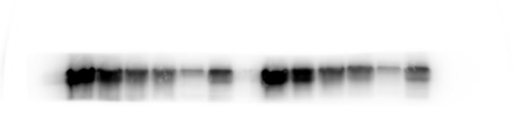

P-Tau Repeat one:

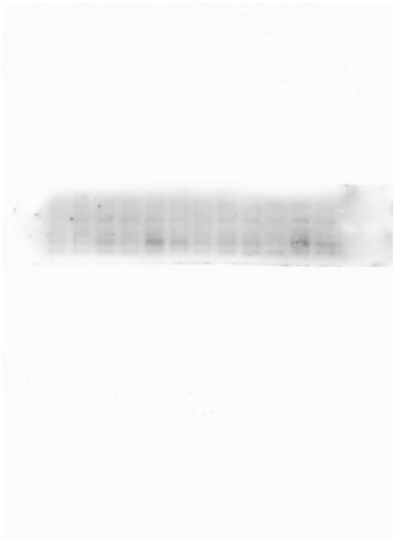

P-Tau Repeat two:

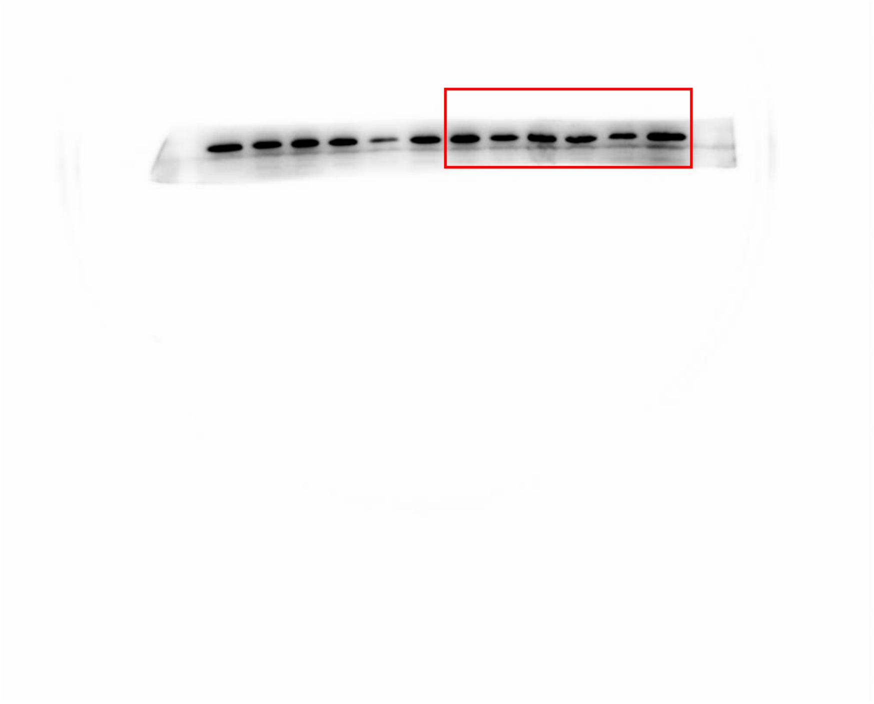

Fig S3 gapdh- full unedited gel for Figure 3

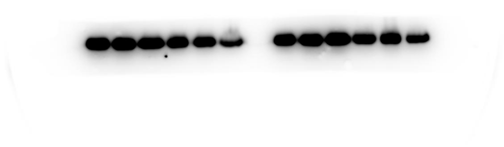

gapdh- Repeat one:

gapdh- Repeat two:

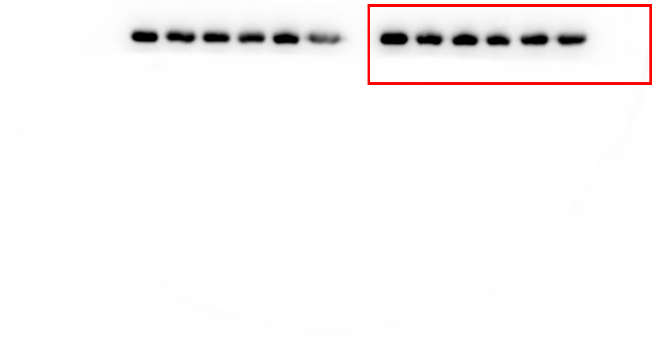

Fig.S4 gapdh--full unedited gel for Figure 4A

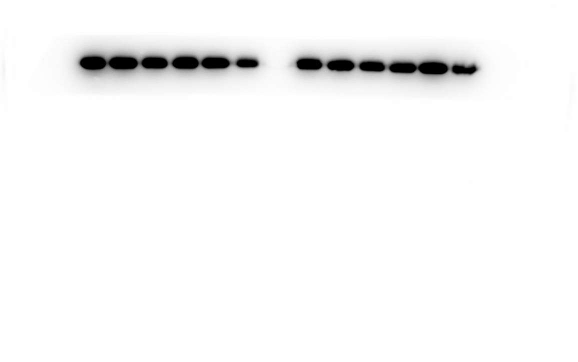

gapdh- Repeat one:

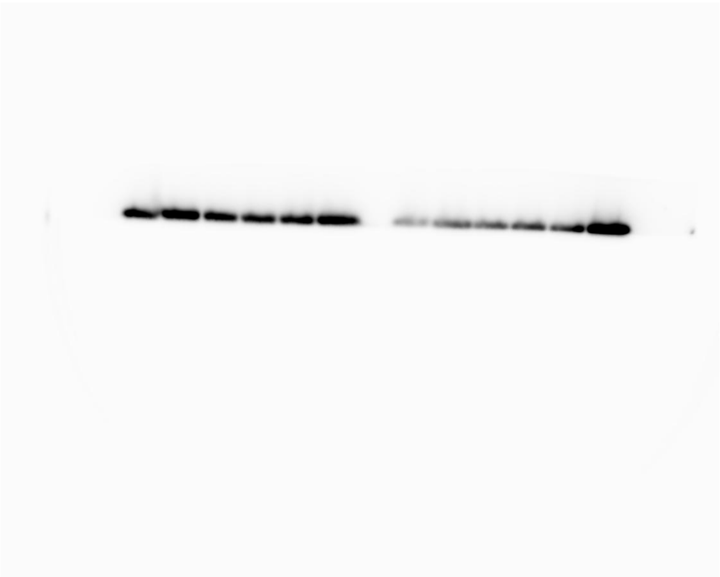

gapdh- Repeat two:

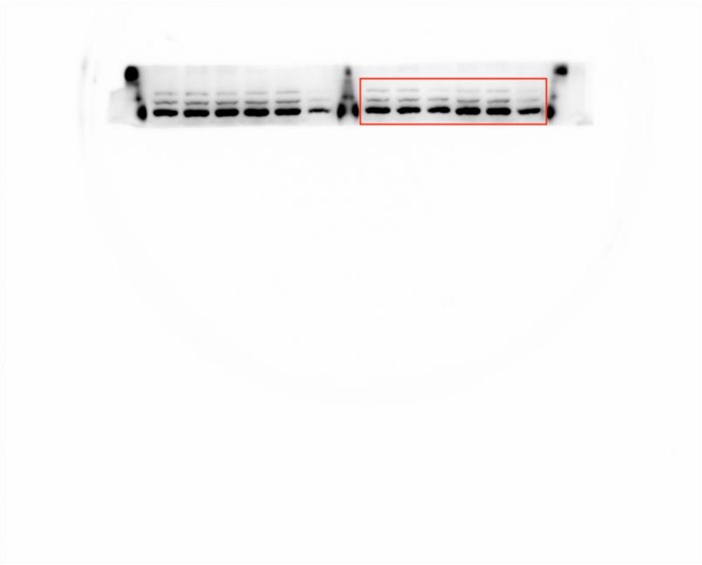

Fig.S5 Nrf2-full unedited gel for Figure 4

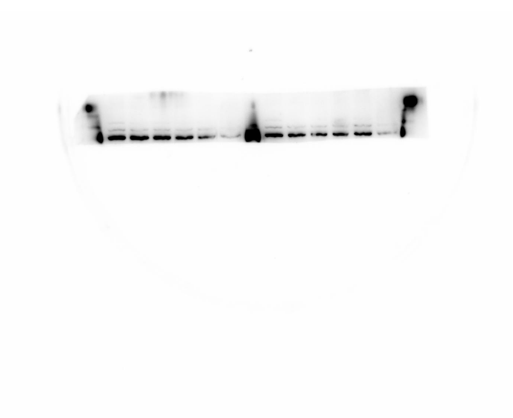

Nrf2- Repeat one:

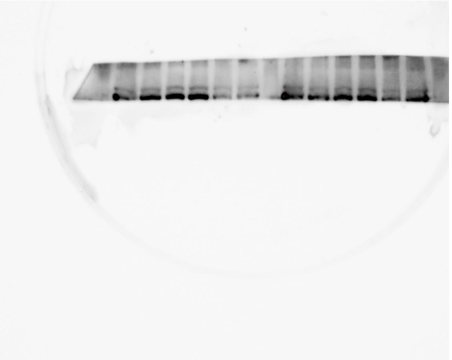

Nrf2- Repeat two:

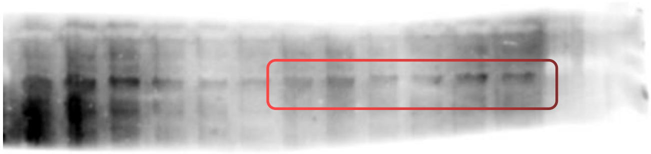

Fig.S6 keap1-full unedited gel for Figure 4

keap1- Repeat one:

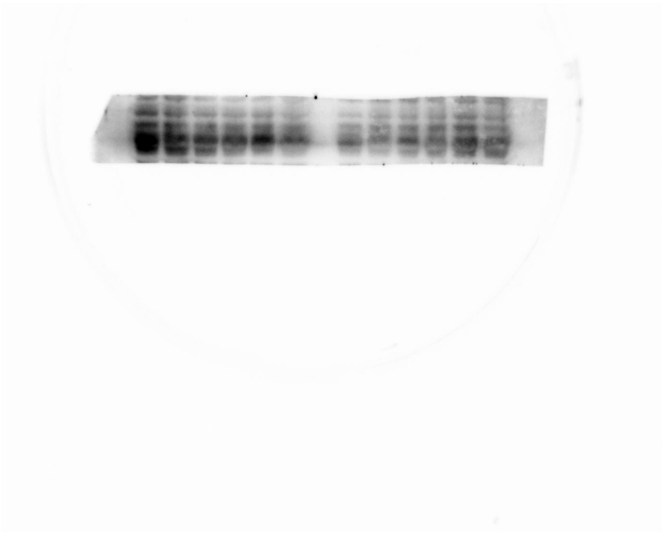

keap1- Repeat two: 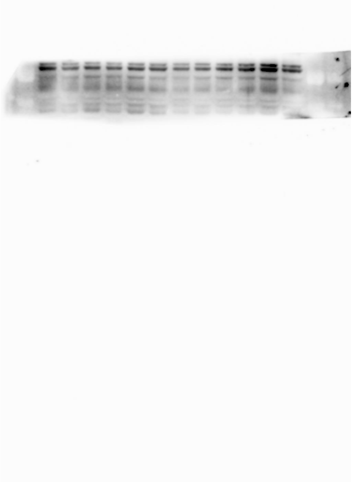

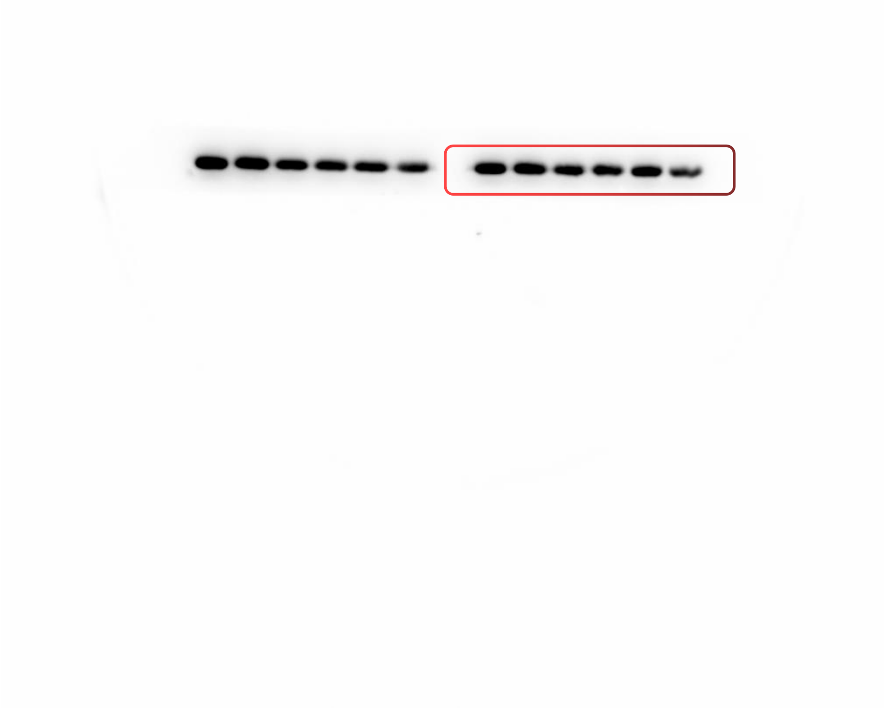

Fig.S7 gapdh--full unedited gel for Figure 4D

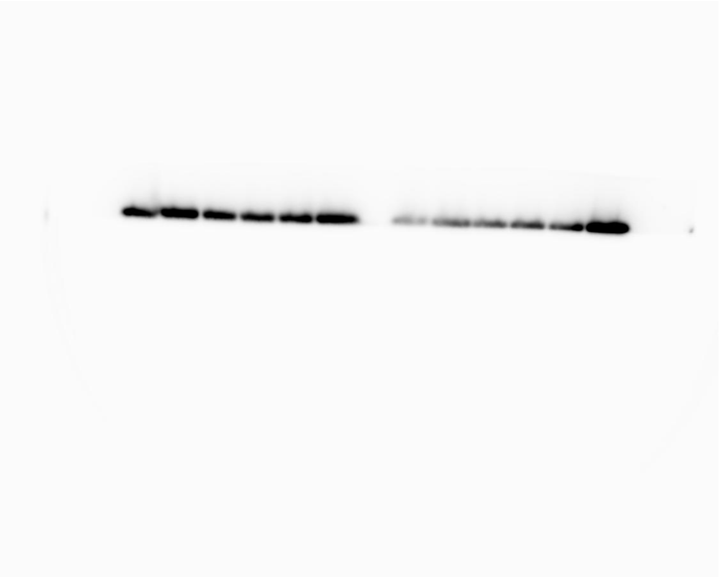

gapdh- Repeat one:

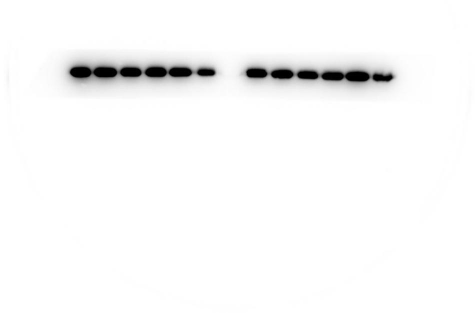

gapdh- Repeat two:

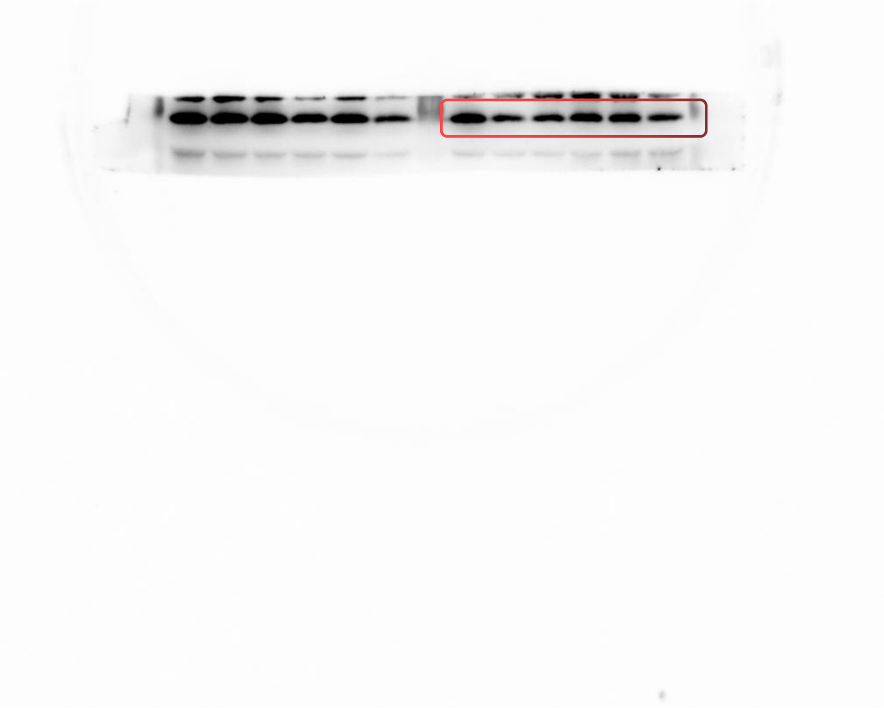

Fig.S8 P62--full unedited gel for Figure 4

P62- Repeat one:

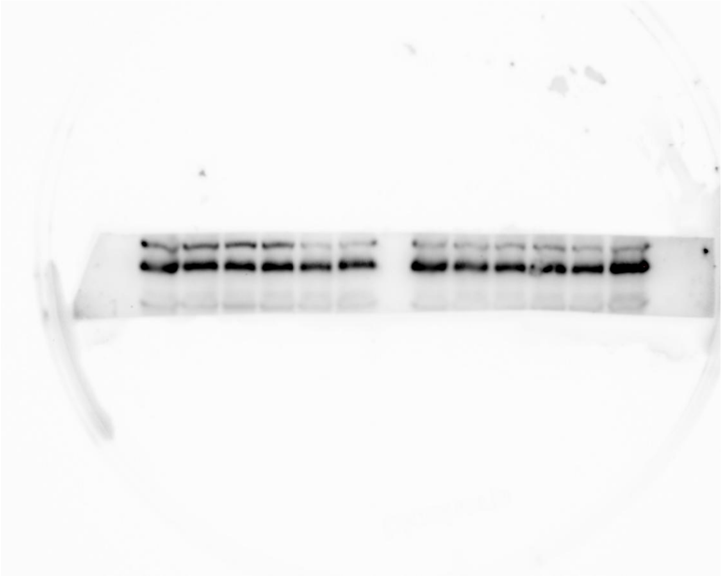

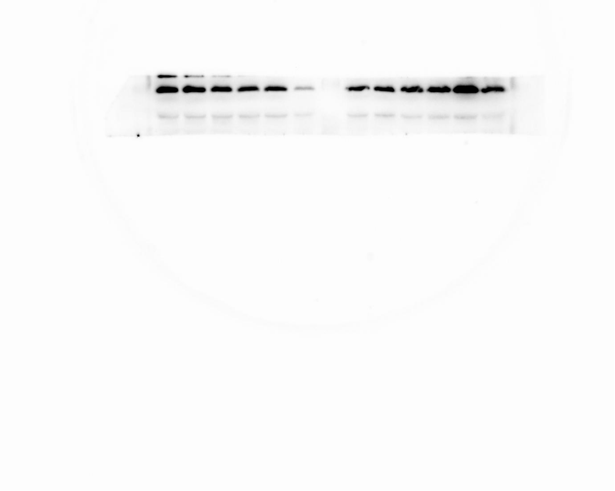

P62- Repeat two:

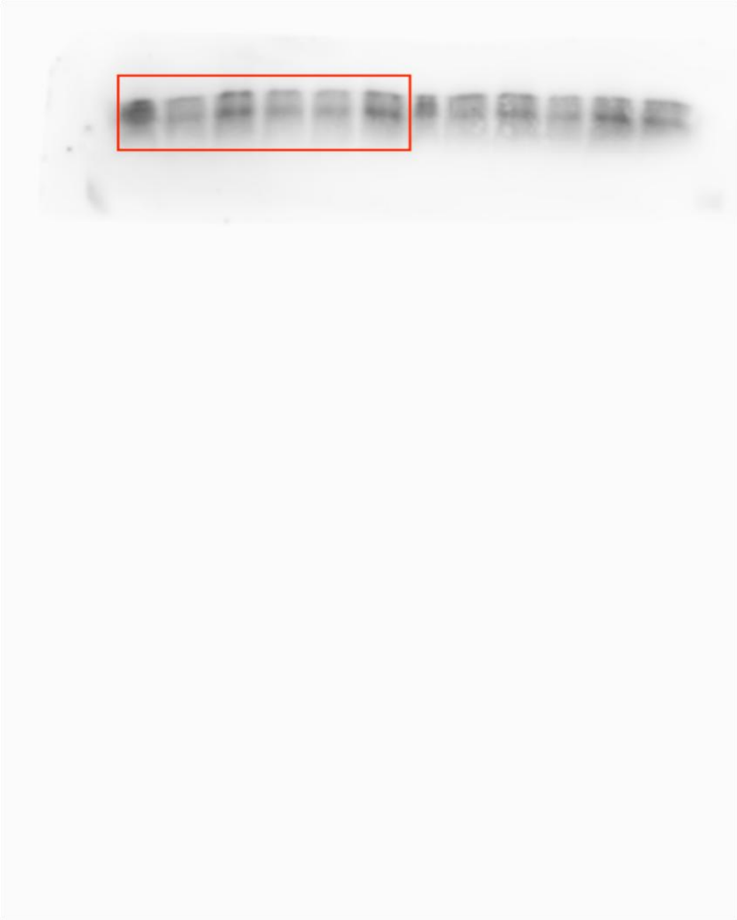

Fig.S9 LC3_full unedited gel for Figure 4

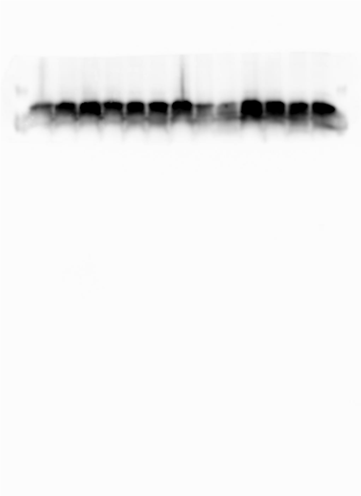

LC3- Repeat one:

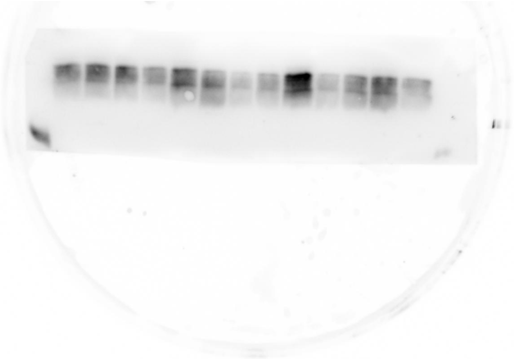

LC3- Repeat two:

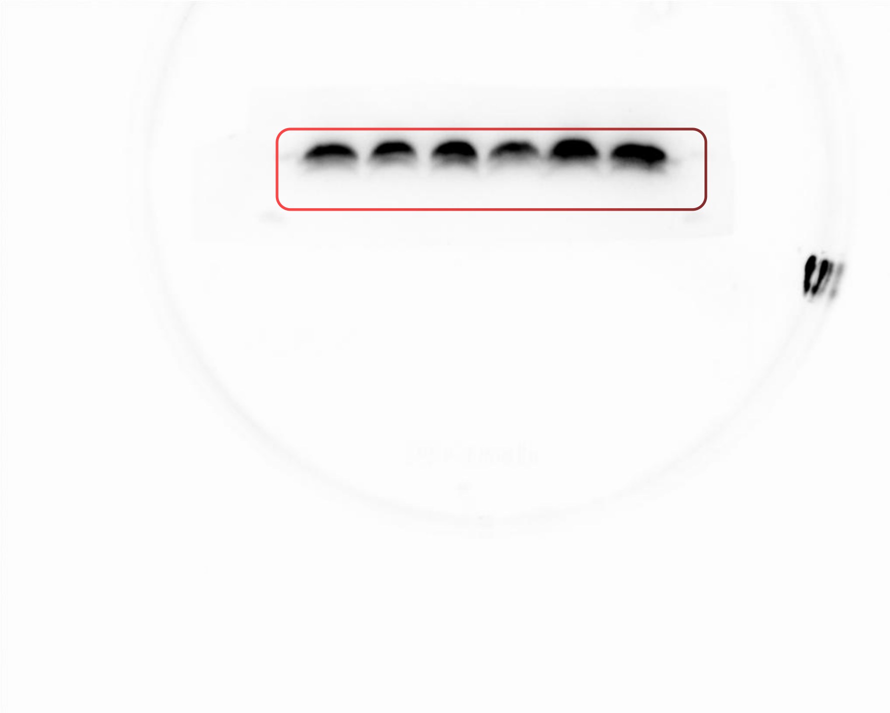

Fig.S10 gapdh-full unedited gel for Figure 6

gapdh- Repeat one:

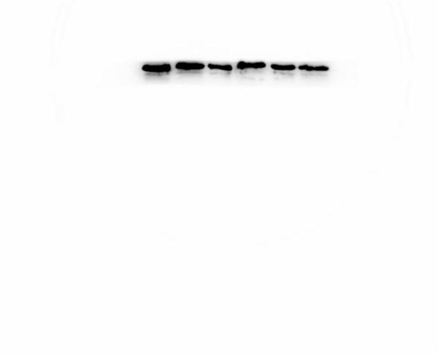

gapdh- Repeat two:

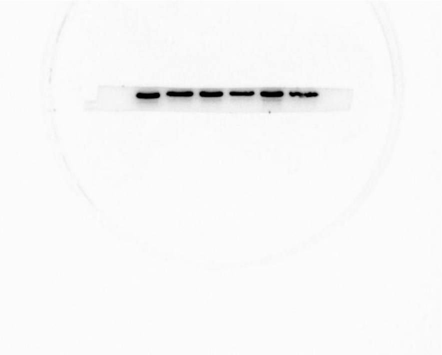

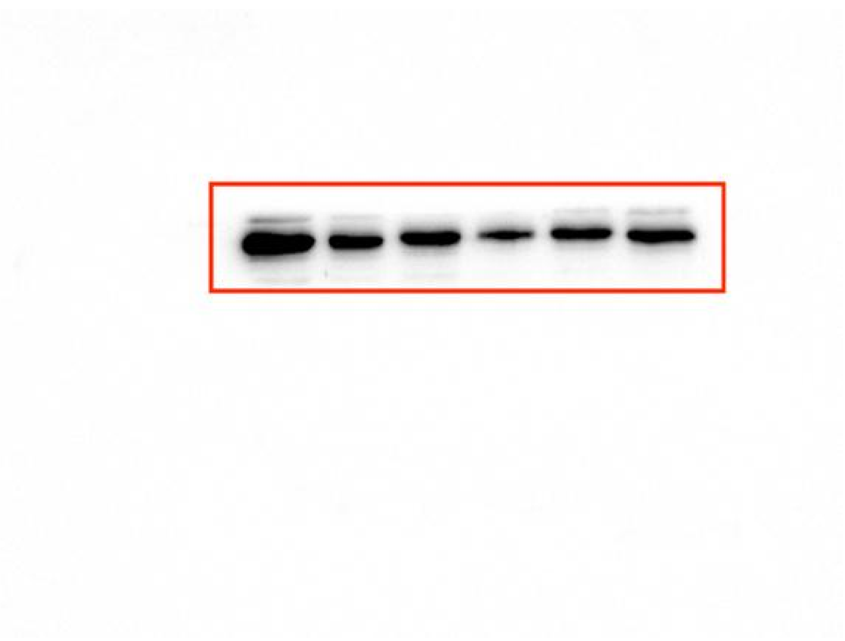

Fig.S11 keap1-full unedited gel for Figure 6

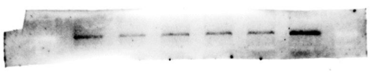

keap1- Repeat one:

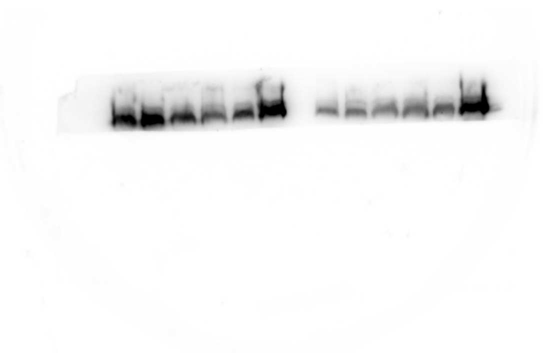

keap1- Repeat two:

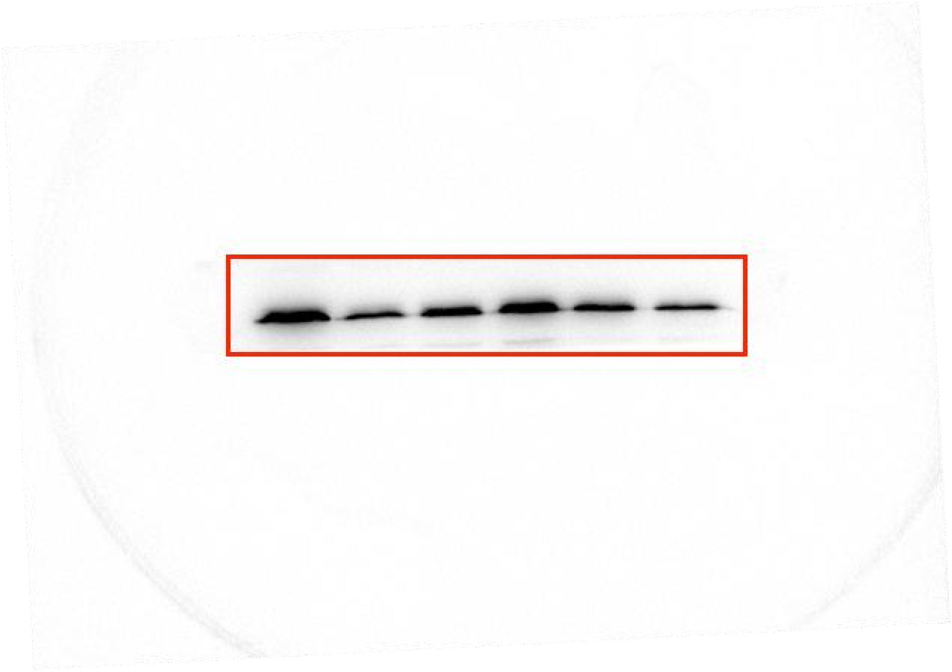

Fig.S12 Nrf2 -full unedited gel for Figure 6

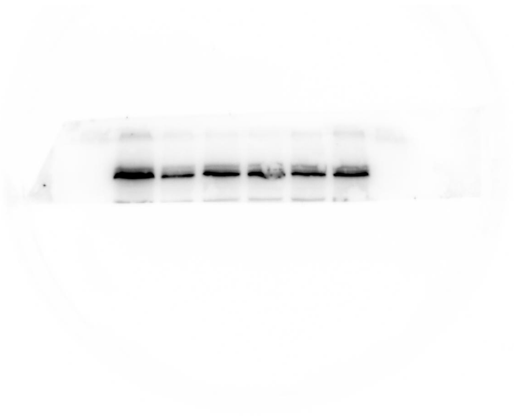

Nrf2- Repeat one:

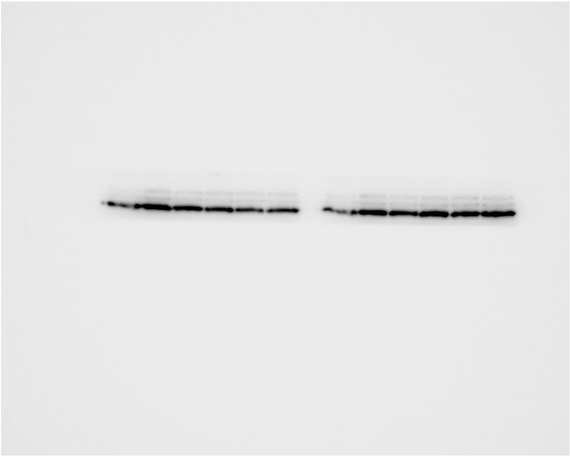

Nrf2- Repeat two:

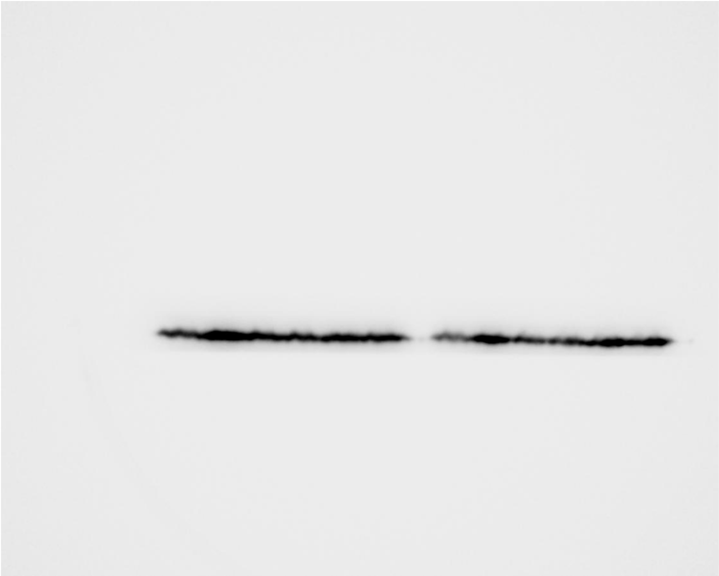

gapdh-Repeat two:

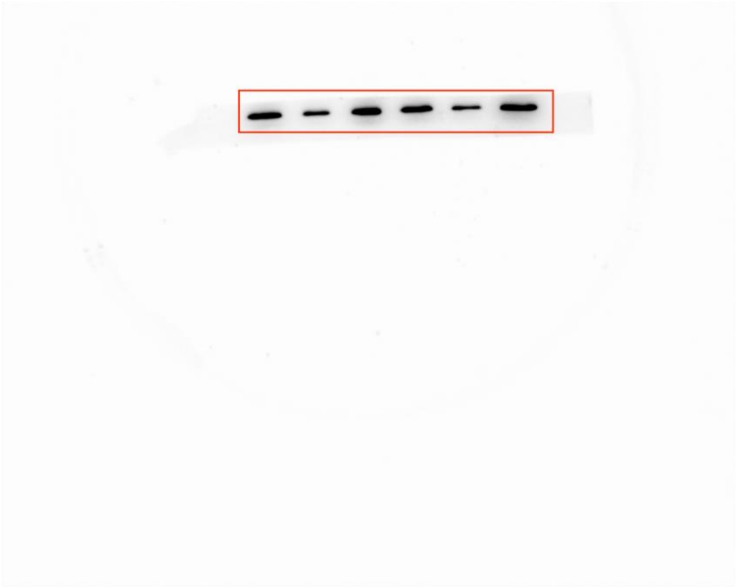

Fig.S13 p62 -full unedited gel for Figure 6

p62- Repeat one:

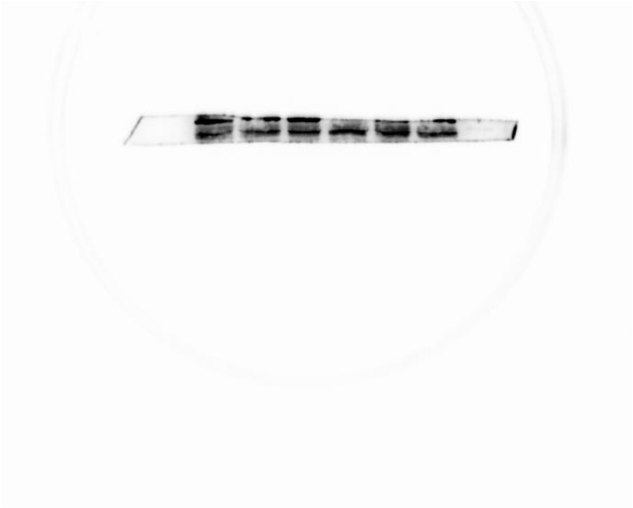

p62- Repeat two:

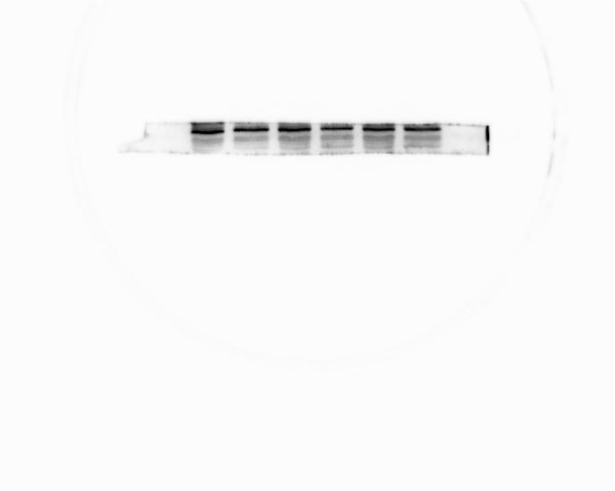

P-Tau- Repeat one:

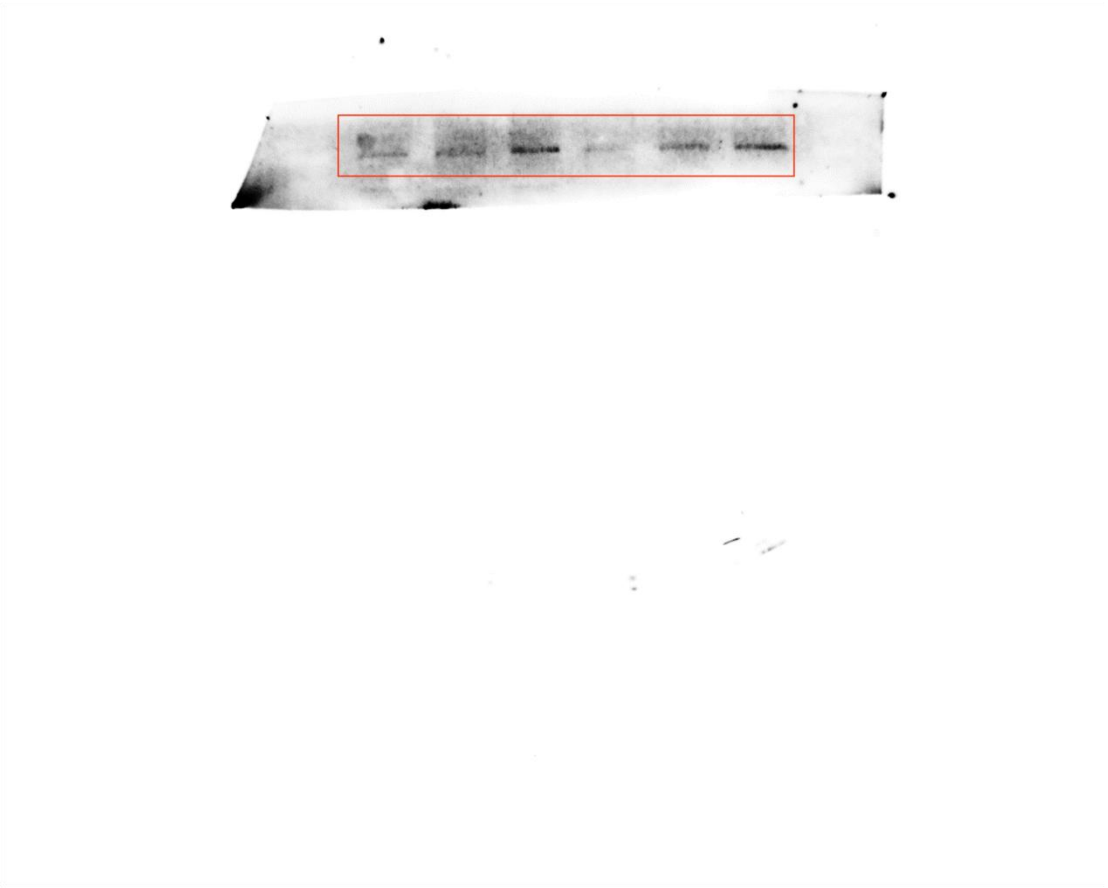

Fig.S14 p-Tau-full unedited gel for Figure 6

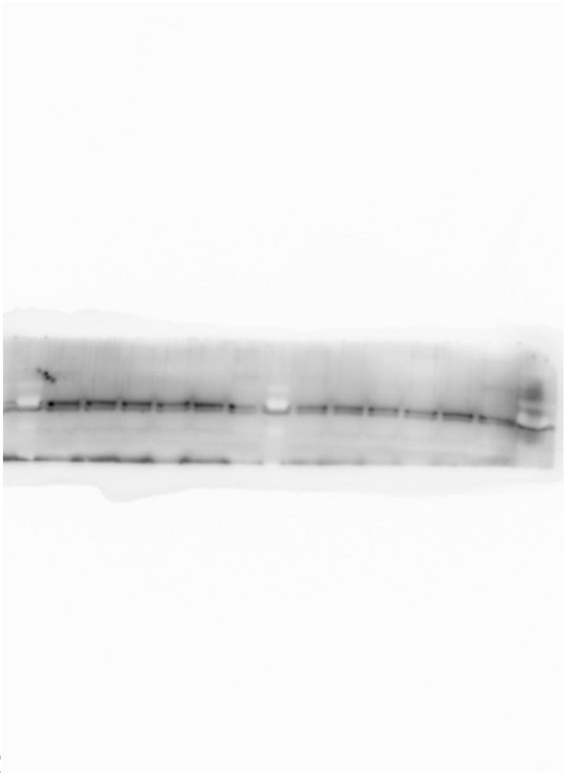

P-Tau- Repeat one:

gapdh-repeat one:

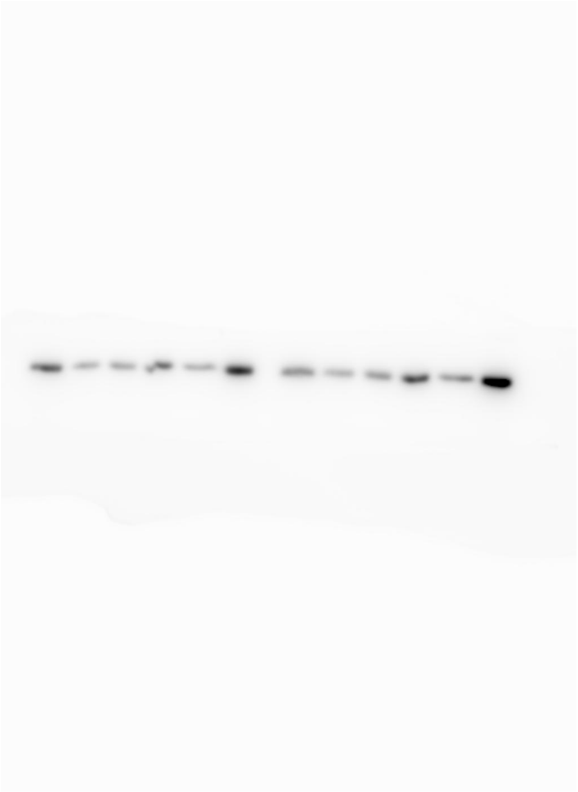

Supplement: Supplementary file 2 — Supplementary Material 2 [file 12906_2023_4290_MOESM2_ESM.pdf]
